# A Data Integration Approach to Mapping OCT4 Gene Regulatory Networks Operative in Embryonic Stem Cells and Embryonal Carcinoma Cells

**DOI:** 10.1371/journal.pone.0010709

**Published:** 2010-05-21

**Authors:** Marc Jung, Hedi Peterson, Lukas Chavez, Pascal Kahlem, Hans Lehrach, Jaak Vilo, James Adjaye

**Affiliations:** 1 Molecular Embryology and Aging Group, Department of Vertebrate Genomics, Max-Planck Institute for Molecular Genetics, Berlin, Germany; 2 Institute of Molecular and Cell Biology, University of Tartu, Tartu, Estonia; 3 Quretec Ltd., Tartu, Estonia; 4 EMBL - European Bioinformatics Institute, Cambridge, United Kingdom; 5 Institute of Computer Science, University of Tartu, Tartu, Estonia; Harvard Medical School, United States of America

## Abstract

It is essential to understand the network of transcription factors controlling self-renewal of human embryonic stem cells (ESCs) and human embryonal carcinoma cells (ECs) if we are to exploit these cells in regenerative medicine regimes. Correlating gene expression levels after RNAi-based ablation of OCT4 function with its downstream targets enables a better prediction of motif-specific driven expression modules pertinent for self-renewal and differentiation of embryonic stem cells and induced pluripotent stem cells.

We initially identified putative direct downstream targets of OCT4 by employing CHIP-on-chip analysis. A comparison of three peak analysis programs revealed a refined list of OCT4 targets in the human EC cell line NCCIT, this list was then compared to previously published OCT4 CHIP-on-chip datasets derived from both ES and EC cells. We have verified an enriched POU-motif, discovered by a *de novo* approach, thus enabling us to define six distinct modules of OCT4 binding and regulation of its target genes.

A selection of these targets has been validated, like NANOG, which harbours the evolutionarily conserved OCT4-SOX2 binding motif within its proximal promoter. Other validated targets, which do not harbour the classical HMG motif are USP44 and GADD45G, a key regulator of the cell cycle. Over-expression of GADD45G in NCCIT cells resulted in an enrichment and up-regulation of genes associated with the cell cycle (*CDKN1B*, *CDKN1C*, *CDK6* and *MAPK4*) and developmental processes (*BMP4*, *HAND1*, *EOMES*, *ID2*, *GATA4*, *GATA5*, *ISL1* and *MSX1*). A comparison of positively regulated OCT4 targets common to EC and ES cells identified genes such as *NANOG*, *PHC1*, *USP44*, *SOX2*, *PHF17 and OCT4*, thus further confirming their universal role in maintaining self-renewal in both cell types. Finally we have created a user-friendly database (http://biit.cs.ut.ee/escd/), integrating all OCT4 and stem cell related datasets in both human and mouse ES and EC cells.

In the current era of systems biology driven research, we envisage that our integrated embryonic stem cell database will prove beneficial to the booming field of ES, iPS and cancer research.

## Introduction

Human embryonic stem cells (hESCs), derived from the inner cell mass (ICM) of the blastocyst, have the ability to differentiate into all cell types and thus hold great potential for regenerative medicine and studying early development [1]. Human embryonal carcinoma cells (hECs) on the other hand, are derived from non-seminoma cells of a testicular germ cell tumour. Testis germ cell tumors are unique in that the normal germ cell from which the tumor is derived has specific stem cell characteristics that are shared with pluripotent hESCs [2]. The stem cell phenotype of hESCs cells has recently been shown to be maintained by a self-stabilizing network of transcription factors, NANOG, OCT4, and SOX2 [3]. These factors maintain their own and each other's transcriptional level, through combinatorial interactions. They positively regulate genes responsible for the ES cell phenotype whilst repressing transcription of genes required for inducing differentiation.

EC cells may be a useful model in deciphering regulatory networks associated with self-renewal and pluripotency [4], [5], [6], [7]. During ES cell differentiation, self-renewal regulating transcription factors such as OCT4 are down-regulated by epigenetic mechanisms, including DNA methylation [8]. Ablation of OCT4 function in human ES cells leads to differentiation into trophectoderm [9] whereas in EC cells it also induces differentiation, but not to the trophectoderm lineage [6]. So in both cell types OCT4 functionality and gene regulatory networks are required for maintaining self-renewal.

OCT4 (also known as POU5F1) was first isolated from mouse ES cells based on its ability to bind the octamer sequence “ATGCAAAT” [10]. During embryogenesis, OCT4 is expressed in primordial germ cells, oocytes, preimplantation embryos and then restricted to the inner cell mass of the blastocyst [11], [12], [13].

Several downstream targets of OCT4 in human ES [3] as well as EC cell lines [14] and mouse ES cells [15] have been identified using ChIP-on-Chip techniques. Interestingly, an inter- and intra- species (ES/EC) comparison of putative OCT4 targets resulted in a rather small overlap of common targets. This, in part, maybe explained by the different platforms and analysis tools employed in these studies.

In order to study gene regulation of transcription factors and their direct targets, it is essential to correlate ChIP-on-chip assays to gene knockdown experiments, specific for the transcription factor under investigation. RNAi-based *OCT4* knockdowns have been performed with NCCIT cells [6] and for the hESC line H1 [9]. In mouse ES cells Loh *et al*. performed RNAi-based knockdowns for *Oct4* and *Nanog* and compared the differential expression pattern with potential binding sites of these factors, using a ChIP-PET approach [15]. For the discovery of Oct4-regulated target genes, Matoba et al. went a step further, combining manipulated *Oct4* levels in mES cells with expression profiling to identify new Oct4 regulated genes [16]. Furthermore, Sharov and colleagues showed that direct target genes for Oct4, Sox2 and Nanog mainly function as activators of downstream gene expression [17]. Finally, applying *Oct4 and Sox2* knockdowns induced by shRNA in mES cells, Walker et al. reported a set of predicted targets of pluripotency [18]. However similar studies for human ES cells are still lacking, given the more restricted use and still inefficient manipulation such as transfecting DNA into these cell lines. Thus we opted for the use of the human EC cell line NCCIT and compared the data generated with existing data related to hES cells [3] in order to find common direct OCT4 target genes, which contribute to the maintenance of pluripotency and self-renewal in both cell types. To achieve this aim, we performed ChIP-on-Chip, experiments using OCT4 antibody and NCCIT cells to obtain a dataset related to OCT4-bound regions close to the transcription start sites of target genes and expanded the complex network regulated by OCT4. In this study, we have integrated our datasets with existing related datasets from both human and mouse ES and EC cells to generate an Embryonic Stem Cell Database (ESCDb). This tool enables rapid and convenient access and comparisons between published datasets related to embryonic stem cell biology.

## Results

### Quality control of OCT4 bound genomic fragments

**Figure 1:**
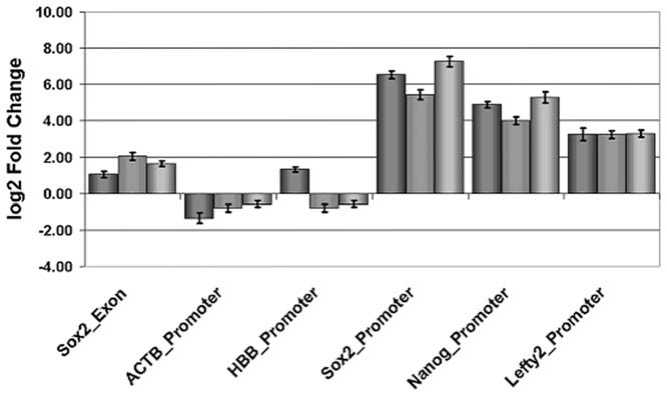
Validation of selected binding sites. Real-time PCR showing relative enrichment values for all 3 biological replicates after amplification. 5′proximal promoter regions were selected for primer sites. SOX2-Exon, ACTB-promoter and HBB-promoter were used as negative controls.

Prior to hybridising the samples onto the NimbleGen-promoter array we performed ChIP-RT-PCR experiments to compare the amplified input (control) DNA with that of OCT4-bound DNA in order to assess the quality of the samples. To achieve this, primers flanking the OCT4-SOX2 binding motifs within the promoter of established OCT4 downstream target genes such as *NANOG*, *SOX2*, *LEFTY2* and *FGF2* [3] were used for the assay. We confirmed a relative enrichment of at least 2-fold for all 3 biological replicates (Fig. 1). Several exon and promoter regions lacking the OCT4 binding site were used as controls.

### Global data analysis

We compared the targets identified by three independent peak analysis programs, including MA2C, TAMALPAIS and an in-house developed peak analysis tool for ratio distribution dependent interval analysis, referred to as brute-force [19]. MA2C [20] and TAMALPAIS [21] are publicly available. TAMALPAIS was used for the promoter analysis as it assumes that only a small fraction (<5%) of probes on an array harbours binding sites of transcription factors (personal communication). A paucity of binding sites has also been observed in other OCT4 ChIP-on-Chip experiments [3], [14] where less than 5% of target genes had the OCT4 binding motif. Five of the six arrays used showed raw correlation coefficients (Cy3 vs Cy5) in the range of 0.91–0.94 with correlation coefficients always slightly higher after applying quantile normalization. The complete results of the quality control and sample images of the hybridisations can be found in Document S1.

**Figure 2:**
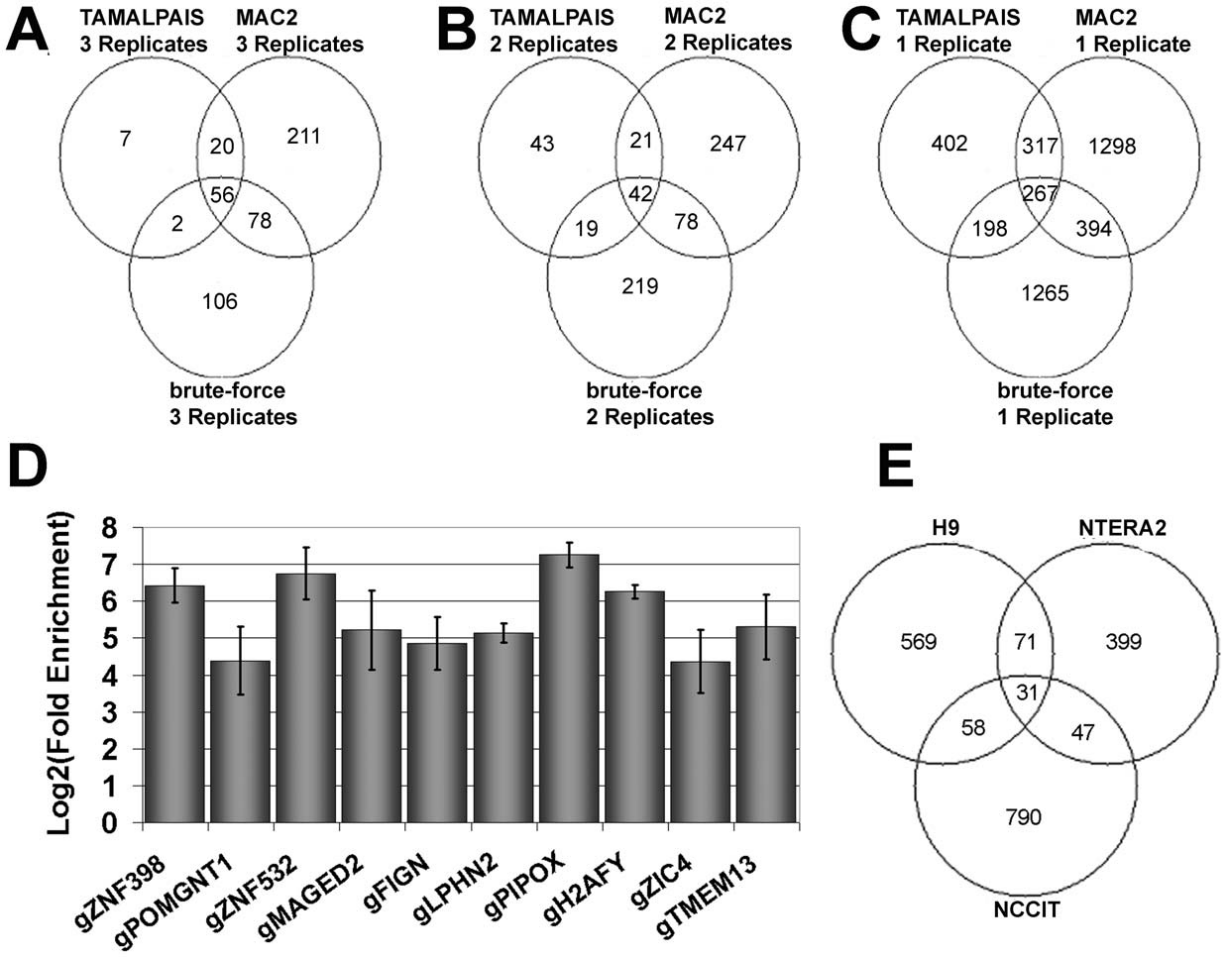
Influence of peak finding algorithms on binding sites. Venn diagrams, illustrating the overlaps between different peak analysis programs. A–C: sorted replicate-wise. D: Real-time PCR validation, showing relative enrichment values for 2 non-amplified biological replicates. Ten randomly chosen peak regions, identified by our peak analysis were chosen for this analysis. ZNF398, POMGNT1, ZNF532 and MAGED2 were identified by all three algorithms. FIGN and LPHN2 were only detected by brute-force. PIPOX and H2AFY were only detected by MA2C. TMEM139 and ZIC4 were only detected by TAMALPAIS. E: Venn diagram, showing the overlap between different cell lines- NCCIT, this study, H9 [3] and NTERA2 [13].

Comparing the three different peak analysis programs, we noticed a significant number of targets were identified exclusively by one program, for peaks detected in up to 3 biological replicates (Fig. 2A–C). This was in accordance with a previous study performed by Johnson et al. showing that the variation in performance between labs, protocols, and algorithms within the same array platform was greater than the variation in performance between array platforms [22]. We considered each program equally for the purpose of peak finding and reasoned that a peak identified by three separate programs in each replicate was equivalent to a peak identified by one program in three biological replicates. We validated 13 ChIP-on-Chip targets by ChIP-real time PCR analysis (Fig. 2D).

**Figure 3:**
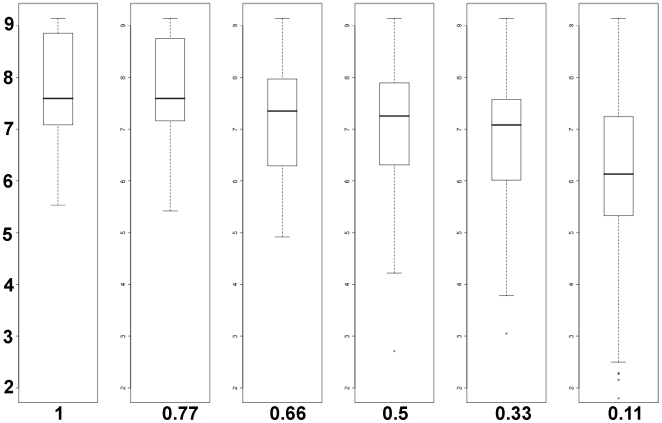
Correlation between octamer motifs and peak score values. Boxplot, showing the distribution of the quality of octamer motifs in relation to our defined peak score. For a peak-score of 0.5, half of the motifs will have a motif-score of 7.3 and above. The average motif score will decrease slightly for a peak score of 0.33 and a significant drop in the motif score can be perceived for a peak score of 0.11.

Early studies in mouse showed that a strong enhancer element for OCT4 binding is the octamer motif [10]. Thus, based on this algorithm, we wondered what the correlation of octamer motifs (which we will refer to as the OCT4 motif) and the peak score value would be. As seen in Figure 3, 50% of all potential octamer motifs fall within peak scores starting at 0.5. The median for the motif scores was 7.3 and was used as a threshold for subsequent motif analysis.

### Downstream targets of OCT4

**Figure 4:**
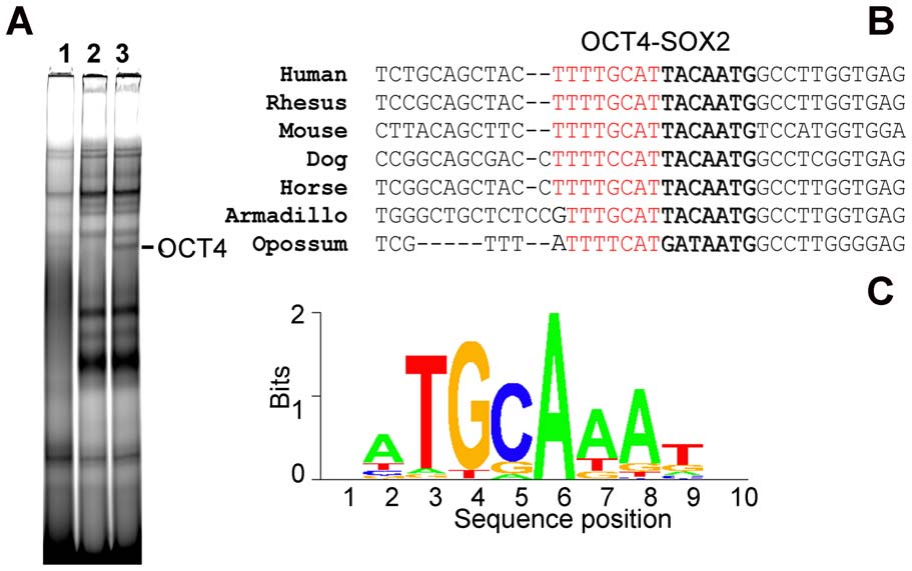
The NANOG promoter harbours an evolutionary conserved binding site. The conserved binding site is shown for OCT4 (red) and SOX2 (bold). A: Bandshift showing a supershift with OCT4 antibody, using NCCIT-derived nuclear extracts and a Cy5 labelled probe in the 5′region of the NANOG promoter bearing the OCT4-SOX2 motif. Binding specificity was tested using oligonucleotide competitors. 1) 20-fold excess of unlabelled competitor. 2) Supershift with OCT4 (sc9081) antibody. 3) Nuclear extract with Cy5-labelled probe. B: Alignment of the OCT4-SOX2 binding sequence in multiple species. C: Bitscore model of the re-constructed OCT4 PWM. Note that the OCT4 PWM sequence is presented in the opposite strand with respect to the sequence shown in (B) above.

The three key pluripotency-regulating genes *OCT4*, *SOX2* and *NANOG* were identified as targets with the highest peak scores. In order to define a threshold for the scores obtained, we defined an OCT4 motif, based on the targets obtained by the three distinct programs (Fig. 4C). We then correlated the genes corresponding to each score with known OCT4 target genes [3], [14], [15].

There was a significant enrichment for OCT4 motifs for a peak score of 0.44 and above. For a peak score of 0.33 and above, 15 of the 16 OCT4 target genes were previously reported as having critical roles in both mouse and human ES cells [23]. Using a threshold of 0.33 resulted in 927 Refseq DNA IDs, which is close to the number of OCT4 targets found in the H9 cell line (729) and almost twice as much as detected with the NTERA2 cell line (548). Comparing the Refseq DNA identifiers from the OCT4 ChIP-on-chip targets with another EC cell line NTERA2 [14] and with a human ES cell line H9 [3] (Fig. 2E), we uncovered a set of 31 targets amongst which are both positively regulated (including OCT4, SOX2 and NANOG) and negatively regulated genes (Table S1). Notably, this list contains a significant enrichment of developmental factors (4,4E^−8^ for multicellular organismal development, using DAVID [24], [25]).

**Figure 5:**
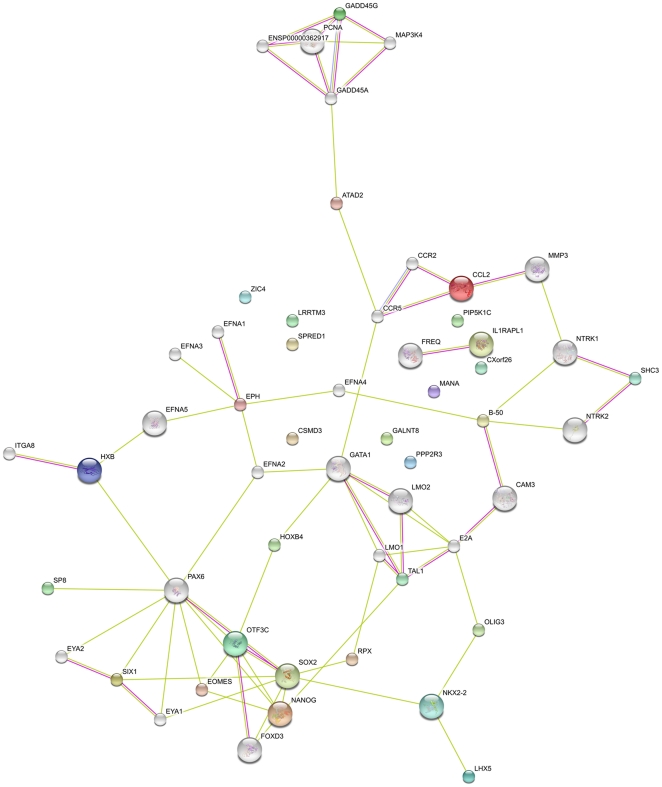
A gene regulatory network based on the 31 genes common in OCT4 ChIP-on-Chip targets derived from NCCIT, NTERA2 and H9 cells. GADD45G was also included in this analysis. The network was generated using the web-based program STRINGS [27]). Pink lines: connectivity based on experimental evidence. Green lines: connectivity based on text mining.

A functional annotation of 46 genes for which peaks have been identified in H9 as well as in NCCIT cells using g:profiler [26], identified genes contributing to neural crest cell development, developmental processes, with an enrichment of genes involved in DNA dependent regulation of transcription. Additionally, we performed a functional annotation of these genes, and the most stringent annotations (p-value <0.01) were homeobox, transcriptional repressors and activators, neuronal differentiation and segmentation. For homeobox-containing proteins, 17 out of the 31 specific targets identified in NCCIT cells, were detected as OCT4 targets in the human ES cell line- H9 as well (Table 1). To determine if these genes potentially exist as an OCT4-gene regulatory network, we submitted this list of genes to the STRINGS network analysis tool [27]. The resulting network (Fig. 5) consisted of a distinct self-renewal cluster composed of NANOG, SOX2, FOXD3, OCT4 (OTF3C) and differentiation-inducing network clusters regulated by transcription factors such as NKX2-2, OLIG3, LHX5, HOXB4 and GATA1, which are themselves negatively regulated by OCT4 [9].

**Table 1:** Examples of Homeodomain containing genes bound by OCT4 in NCCIT and H9 cells [3].

HGNC symbol	Description	RefSeq DNA ID	Occupied by OCT4 in H9
TPRX1	Tetra-peptide repeat homeobox protein 1	NM_198479	
HOXB4	Homeobox protein Hox-B4	NM_024015	**+**
HOXC10	Homeobox protein Hox-C10	NM_017409	
TGIF2LX	Homeobox protein TGIF2LX (TGFB-induced factor 2-like protein, X-linked) (TGF(beta)induced transcription factor 2-like protein) (TGIF-like on the X)	NM_138960	
ADNP	Activity-dependent neuroprotector homeobox protein (Activity-dependent neuroprotective protein)	NM_015339	
SIX1	Homeobox protein SIX1 (Sine oculis homeobox homolog 1)	NM_005982	**+**
OTX2	orthodenticle homeobox 2	NM_021728	
MEIS2	Homeobox protein Meis2 (Meis1-related protein 1)	NM_172315	
MEIS1	Homeobox protein Meis1	NM_002398	**+**
ISL1	Insulin gene enhancer protein ISL-1 (Islet-1)	NM_002202	**+**
LHX5	LIM/homeobox protein Lhx5 (LIM homeobox protein 5)	NM_022363	**+**
PITX3	Pituitary homeobox 3 (Homeobox protein PITX3)	NM_005029	
HOXB6	Homeobox protein Hox-B6 (Hox-2B) (Hox-2.2) (HU-2)	NM_156037	**+**
HOXB1	Homeobox protein Hox-B1 (Hox-2I)	NM_002144	**+**
PHOX2A	Paired mesoderm homeobox protein 2A (Paired-like homeobox 2A) (Aristaless homeobox protein homolog) (ARIX1 homeodomain protein)	NM_005169	
PITX2	Pituitary homeobox 2 (RIEG bicoid-related homeobox transcription factor) (Solurshin) (ALL1-responsive protein ARP1)	NM_153426	
HESX1	Homeobox expressed in ES cells 1 (Homeobox protein ANF) (hAnf)	NM_003865	**+**
GSC	Homeobox protein goosecoid	NM_173849	**+**
HOXA3	Homeobox protein Hox-A3 (Hox-1E)	NM_030661	**+**
POU5F1	POU domain, class 5, transcription factor 1 (Octamer-binding transcription factor 3) (Oct-3) (Oct-4)	NM_002701	**+**
ZHX3	Zinc fingers and homeoboxes protein 3 (Zinc finger and homeodomain protein 3) (Triple homeobox protein 1)	NM_015035	
MEOX2	Homeobox protein MOX-2 (Mesenchyme homeobox 2) (Growth arrest-specific homeobox)	NM_005924	
TGIF2	Homeobox protein TGIF2 (5′-TG-3′-interacting factor 2) (TGF(beta)-induced transcription factor 2) (TGFB-induced factor 2)	NM_021809	**+**
NANOG	Homeobox protein NANOG (Homeobox transcription factor Nanog) (hNanog)	NM_024865	**+**
TSHZ1	Teashirt homolog 1 (Serologically defined colon cancer antigen 33) (Antigen NY-CO-33)	NM_005786	
NKX2-2	Homeobox protein Nkx-2.2 (Homeobox protein NK-2 homolog B)	NM_002509	**+**
BARX2	Homeobox protein BarH-like 2	NM_003658	
HOXD13	Homeobox protein Hox-D13 (Hox-4I)	NM_000523	
HOXD11	Homeobox protein Hox-D11 (Hox-4F)	NM_021192	**+**
HOXD8	Homeobox protein Hox-D8 (Hox-4E) (Hox-5.4)	NM_019558	
HIPK1	Homeodomain-interacting protein kinase 1 (EC 2.7.11.1)	NM_181358	
GBX2	Homeobox protein GBX-2 (Gastrulation and brain-specific homeobox protein 2)	NM_001485	**+**
PROX1	Prospero homeobox protein 1 (Homeobox prospero-like protein PROX1) (PROX-1)	NM_002763	**+**

### Distinct OCT4 binding modules

To investigate if most of our targets contain an octamer motif, we screened all the peak regions of 497 target genes for OCT4 motifs, using a peak-score of 0.5 and ranked them based on a significance score. Genes with scores of 7.3 and above were defined as potential direct targets of OCT4 as defined above. We then sorted all targets with an OCT4 and a SOX2 motif above the threshold level, resulting in a list of 372 genes. The comparison of this list with the target list from Boyer et al. [3] that had a SOX2 and an OCT4 peak region (332 targets), resulted in an overlap of 293 targets.

**Figure 6:**
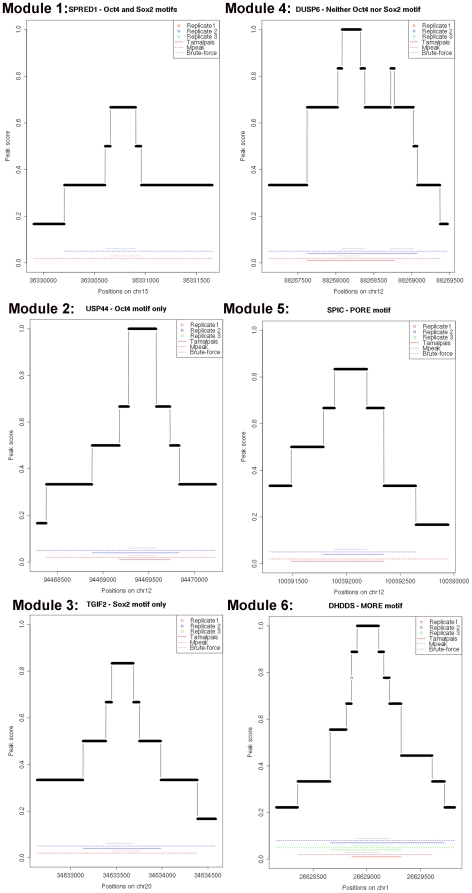
Six distinct OCT4 binding modules. Shown are the peak scores, relative to the overlap between MAC2, TAMALPAIS, the in-house developed algorithm - brute-force [19] and the biological replicates. Peak profiles could be screened for the octamer and SOX2 motifs.

Additionally we were interested in all target genes containing a motif score below 7.3. To investigate if these targets could be regulated by another transcription factor, we scanned these regions with motif matching programs [28], [29], [30]. In addition to OCT4 motifs, we screened our peak regions for the presence of SOX2 motifs, as it is known to form a heterodimer with OCT4. This analysis led to the identification of 6 distinct putative modules of OCT4-binding and transcriptional regulation (Fig. 6, Table S2). In all of these modules we correlated the corresponding genes with their significant 2-fold up- or down-regulated expression upon *OCT4* knockdown in NCCIT cells [6] (Table S3).

### Module 1: OCT4-SOX2 binding motif

This group consists of 39 genes in total. Within this module, *CTGF* and *TXNRD1* were up-regulated whilst *TPST2*, *PAK1* and *NANOG* were down-regulated in *OCT4* depleted NCCIT cells [6]. We validated the binding of OCT4 to the OCT4-SOX2 motif within the proximal promoter of the NANOG gene in NCCIT cells using a bandshift assay (Fig. 4).

### Module 2: OCT4 binding motif but lacking a SOX2 binding motif

**Figure 7:**
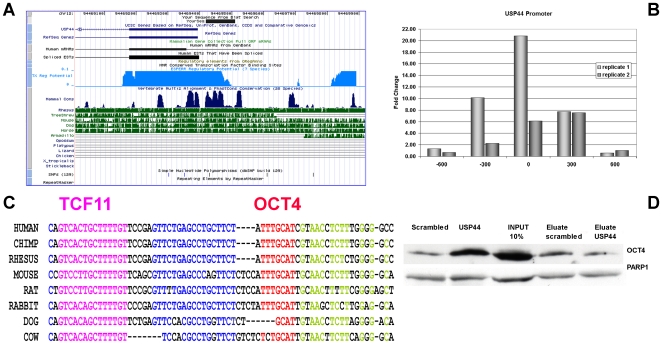
The USP44 promoter harbours the evolutionary conserved OCT4 binding site but lacks the SOX2 motif. A: Sequence containing the conserved POU site as displayed by the UCSC genome browser. B: Real time PCR confirmation of the presence of the OCT4 binding site. Position 0 indicates the conserved region seen in (A). C: Multiple alignments showing evolutionary conservation of the OCT4-bound region. The sequences depicted in blue and green are uncharacterised with respect to transcription factor recognition and binding. D: Western blot analysis of proteins bound to biotinylated oligos representing the promoter fragment shown in (A). The OCT4 antibody shows higher binding intensity to the USP44- specific probe compared to the corresponding scrambled oligo.

This module consists of 122 genes in total, of these *FOXC1*, *RUNX1*, *LGALS3*, *NR2F2*, *CRABP1*, *CAMK2D*, *GFOD1* and *HN1* were up-regulated whilst *GAGE7*, *GAGE8*, *ZNF398*, *USP44* and *DPPA4* were down-regulated in OCT4 depleted NCCIT cells. We also validated the binding of OCT4 to an evolutionary conserved OCT4 motif, which we found in the proximal promoter region of the USP44 gene. A recent publication by Stegmeier reinforced the role of USP44 as an essential enzyme involved in the control of the anaphase promoting complex [31]. As the transcriptional level of *USP44* decreases significantly upon OCT4 knockdown and also in other self renewal perturbation experiments in ES and EC cells [31] we aimed at investigating a possible correlation between OCT4, USP44 and cell cycle control with respect to maintaining self-renewal in these cells. Using the conserved fragment as bait, we could demonstrate an enrichment of OCT4 in a pull-down assay (Fig. 7). Furthermore, we could also confirm the signal obtained by ChIP-real-time-PCR. In addition, we identified a potential binding site for TCF11 within the same conserved region of the USP44 promoter. The transcription factor TCF11, has been implicated in the regulation of antioxidant responses [32] and its function is vital during embryonic development [31].

**Figure 8:**
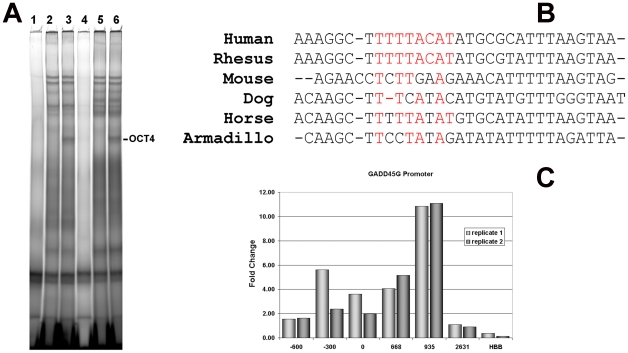
The GADD45G promoter harbours the evolutionary conserved OCT4 binding sites. A: Bandshifts showing supershifts with OCT4 antibody using NCCIT cells derived- nuclear extracts using two probes in the 5′region of the GADD45G promoter containing an OCT4 motif at positions 9–15 (lane 1–3) and 17–23 (lane 4–6) of 31 nucleotides. Lane 3,6: Nuclear extract plus labelled probe. Lane 2,5: same as lanes 3 and 6 but with the addition of OCT4 antibody (sc-9081). Lane 1,4: same as lanes 3 and 6 but with the addition of a 20-fold increase in unlabelled competitor oligo. B: Multi-species alignment of the selected region chosen for the bandshift assay, the conserved OCT4 binding site is highlighted in red. C: Real time PCR confirmation of the presence of the OCT4 binding site. Position 0 indicates the position shown in the alignment in panel 2B.

Another gene harbouring this module in its promoter is *GADD45G*, a regulator of the cell cycle at the G2/M transition [33] and also recently identified as a putative OCT4/PORE target gene [34]. Binding activity was not detected in our ChIP-on-chip target list but was detected in the Boyer dataset [3]. Furthermore, it has been shown to be one of the earliest OCT4-responsive target genes [17] and was significantly upregulated in our OCT4 knockdown experiments. To confirm *GADD45G* as a bona fide direct target of OCT4, we performed a ChIP-real-time-PCR reaction, and confirmed the fold enrichment immediately flanking the OCT4 motif compared to neighbouring sites. We obtained fold changes of above 2 for two replicates with a peak approximately 1 kb upstream of the OCT4 motif (Fig. 8). For additional independent confirmation of binding, we performed a bandshift assay using two oligos flanking the core OCT4 motif (Fig. 8A). We obtained a supershift with OCT4 antibody for both sets of oligos, thus demonstrating specific binding of OCT4 to this locus.

**Figure 9:**
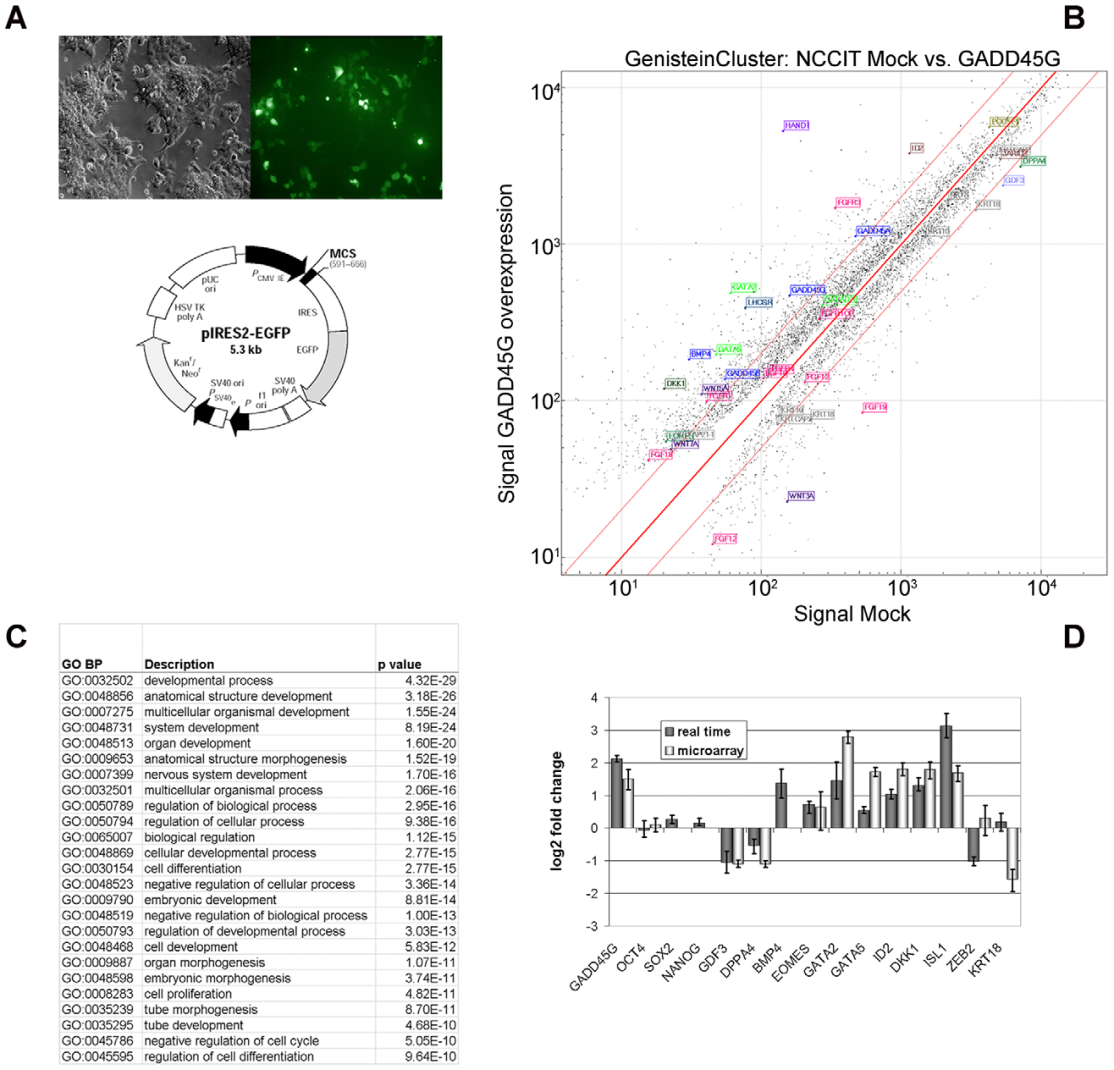
Over-expressing GADD45G in NCCIT cells. A: Presence of GFP expression 48 h post-transfection (left) compared to the phase-contrast image of the cells. The map of the vector used is presented below. B: Scatter plot comparing the transcriptomes of GADD45G transfected cells against cells transfected with the wild-type vector. GADD45G-mediated induction of transcription factors such as *HAND1* (purple), *GATA4* (green), and *ID2* (brown) depicted in boxes. C: Table listing the most significant GO:biological processes related to the up-regulated (>2-fold) genes. D: Real time PCR validation of a selection target genes (NANOG, SOX2 and BMP4 were below detection score 0.01).

As the transcriptional level of *GADD45G* increases significantly (more than 2-fold) upon differentiation of ESC and EC cells as a result of ablating OCT4 function [6], [9], we hypothesised that activation of GADD45G activity would induce loss of self-renewal and hence differentiation of the cells with a concomitant decrease in the expression of OCT4. To test this hypothesis, we cloned the *GADD45G* coding sequence into the pIRES2-eGPF vector and screened for fluorescence as a control for transfection efficiency (Fig. 9A) as good quality antibodies are currently unavailable. RNA was isolated two days post-transfection, and microarray based gene expression analysis carried out (Fig. 9B).

Though morphological changes could not be observed, transcriptional analysis revealed 531 genes with induced expression of 2-fold and higher. Functional annotation analysis revealed a significant enrichment for genes associated with the cell cycle and differentiation processes (Fig. 9B, C, D). A selection of genes were chosen for independent confirmation of expression levels using real-time-PCR. We noted an up-regulation of differentiation associated marker genes, *BMP4*, *HAND1*, *EOMES*, *ID2*, *GATA4*, *GATA5*, *ISL1* and *MSX1* (Fig. 9D). Interestingly, *MSX1* and *MSX2* are known BMP4 downstream target genes [35]. Indeed we could confirm an up-regulation of BMP4 and both genes were highly up-regulated upon OCT4 knockdown in ES and EC cells [6], [9]. *ISL1* is a LIM-homeobox containing gene important for developmental and regulatory function in islet, neural, and cardiac tissue [36].

Although over-expressing GADD45G in NCCIT cells induced up-regulated expression of genes associated with differentiation processes, this was not accompanied by a change in the mRNA or protein levels of OCT4, NANOG and SOX2 at the time point analysed (Data not shown). However, down-regulation of pluripotency associated genes such as *GDF3* [37] and *DPPA4* [38] was observed (Fig. 9D). This result raises the possibility that GADD45G activates transcription of differentiation inducing transcription factors independent of the OCT4, SOX2 and NANOG circuitry. Alternatively, it could be that the increased activity of GADD45G induces rapid suppression of OCT4, SOX2 and NANOG function via for example disrupting posttranslational modifications or protein-protein interaction required for sustaining the self-renewal circuitry. This action probably takes place long before the reduction of the mRNA and protein levels of OCT4, SOX2 and NANOG at least at the time point analysed.

### Module 3: SOX2 binding motif but lacking an OCT4 motif

This set consist of 65 genes in total, of these *EMP1*, *RIN2*, *TNC*, *KLHL5*, *FOXB1*, *PKD1L2*, *GPC6* and *CBR3* were up-regulated whilst *GSPT2*, *HESX1*, *RHCE*, *RHD*, *SFRP2* and *GDF3* were down-regulated in OCT4 depleted NCCIT cells.

### Module 4: SOX2 and OCT4 binding motif not present

**Figure 10:**
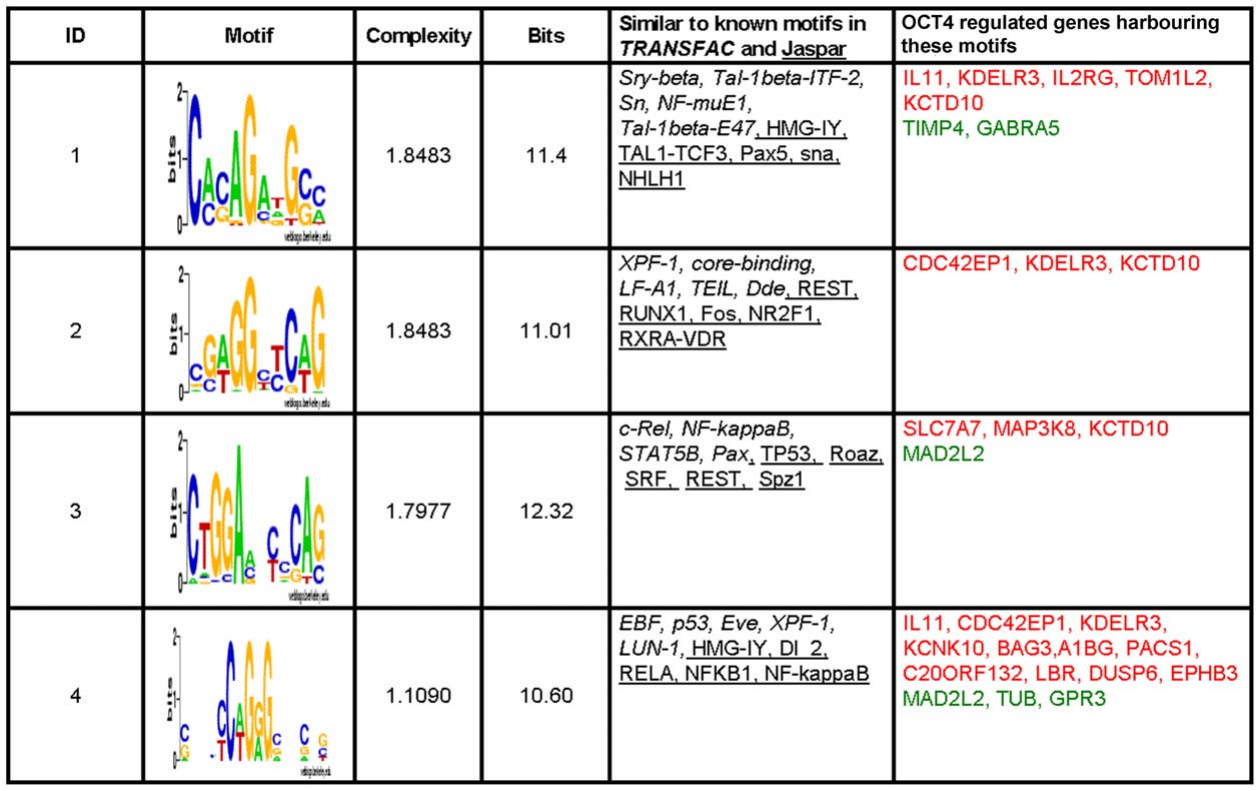
Potential new interaction partners of OCT4. *De novo* motif discovery for genes, identified as OCT4 indirect targets and differentially regulated (2-fold and above) in NCCIT cells but lacking the OCT4 and SOX2 motif within the promoter region analysed. The 4 most significant motifs identified and the potential transcription factor binding sites related to these motifs are displayed. In addition, putative regulated genes harbouring these motifs in their promoter regions shown. Red depicts up-regulated and green down-regulated in response to the ablation of OCT4 activity in ES and EC cells.

This is a very interesting module suggesting that within 3.5 kb upstream and 750 bp downstream of the TSS of the 271 genes identified, OCT4 might be part of a protein complex with yet unknown transcription factor(s) physically contacting the promoter regions of these target genes. Of these genes, *IL1*, *COL4A1*, *PLAU*, *TPM1*, *SYTL2*, *CDC42EP1*, *KDELR3*, *KLNK10*, *H2AFY*, *SLC7A7*, *LGI1*, *BAG3*, *PACS1*, *MAP3K8*, *TOM1L2*, *LBR*, *KCTD10*, *ZFP90*, *EPHB3*, and *WDR1* were up-regulated whilst, *SCGB2A2*, *GABRA5*, *FRAT2*, *RAB25*, *CSPG5*, *MAD2L2*, *SPTBN2*, *C20orf12*, *PHC1*, *MYCN*, *TUB*, *GPR3* and *TIMP4* were down-regulated in OCT4 depleted NCCIT cells. For these regulated genes, we investigated if within the respective promoter regions where putative indirect OCT4 binding activity could be confirmed, one could also detect an enrichment of known transcription factor binding sites by adopting a *de novo* motif discovery approach. Our hypothesis was that some of these sites might recruit OCT4 into a complex, which is not dependent on direct OCT4-DNA interaction for activating or repressing downstream target genes. Using this strategy, we identified four significant motifs predicted to be the binding sites for transcription factors such as REST, TCF3, NR2F1, p53, NF-kB, LF-A1, RUNX1 and PAX5 (Fig. 10).

### Module 5: PORE motif

The PORE sequence (Palindromic Oct factor Recognition Element ATTTGAAATGCAAAT) shown to co-operatively bind two OCT4 molecules was first identified within the first intron of the Osteopontin gene [39]. In our analysis we identified 4 PORE target genes, *ATXN3*, *CIR*, *FLJ16611* and *SPIC*. However, none of these genes were significantly regulated upon knockdown of OCT4 in NCCIT cells.

### Module 6: MORE motif

**Figure 11:**
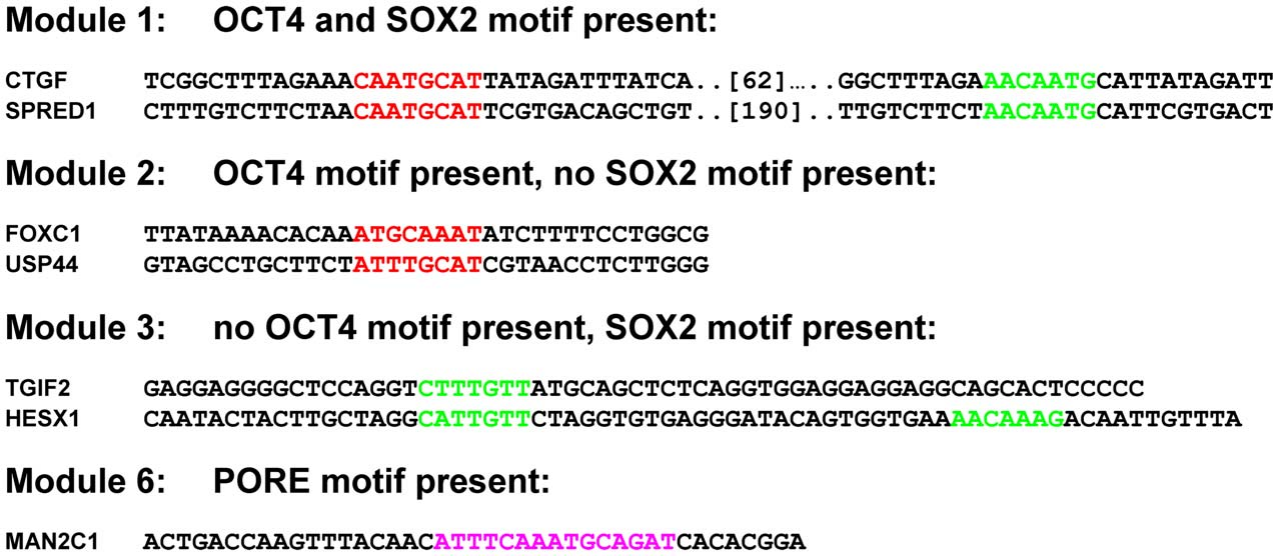
Sequence alignments of selected OCT4-regulated genes under the distinct modules. The OCT4 binding motif is represented in red and that of SOX2 in green.

This motif (More PORE- ATGCATATGCAT) was discovered after the PORE sequence was identified. Like the PORE motif, the MORE sequence also co-operatively binds to two OCT4 molecules [40]. OCT4 targets bearing this motif include *ATPBD4*, *C14orf94*, *CLLU1*, *DHDDS*, *SNX20*, *ORFA17*, *REM2*, *SERPINB7*, *UBE2C* and *GSPT2*. Interestingly *GSPT2*, which encodes a GTP-binding protein that plays an essential role at the G1 to S-phase transition in human cells is also regulated by OCT4 under module 3 (conserved SOX2 binding motif but lacking OCT4). Furthermore, knockdown of OCT4 in NCCIT resulted in a down-regulated expression of *GSPT2* and *UBE2C*. To further describe these modules *in silico* we aligned the sequences under the respective OCT4 binding peak regions of selected genes within each module (Fig. 11).

### Data integration in the form of an Embryonic Stem Cell database

We are in an era of high-throughput functional genomics and systems biology-driven research where large datasets are usually needed and provided as supplementary tables in most publications. Though useful, such tables in isolation are of limited use for making cross-references across other related datasets. Furthermore, as similar approaches have recently been adopted in constructing the HaemAtlas which serves as a reference library for gene expression in human blood cells and as a resource for identifying key genes with roles in blood cell function [41], we have developed a specialized database, which enables rapid and convenient access and comparisons between published datasets related to embryonic stem cell biology to help overcome this shortfall. In order to facilitate the construction of this database, we gathered previously published datasets together with ChIP-on-chip using OCT4 and the NCCIT cell line described in this present work to establish the Embryonic Stem Cell Database (http://biit.cs.ut.ee/escd/). The new database provides easy access to transcription factor binding data together with various perturbation experiments. ESCDb gathers mainly two types of data – chromatin immunoprecipitation array-based data on transcription factor targets and gene specific knockdown of pluripotency associated factors (OCT4, SOX2 and NANOG) as well as growth factor (FGF2) withdrawal and cytokine (BMP4 and ACTIVIN A) stimulation of human ES cells. We have gathered data for mouse and human, and to complement embryonic stem cell experiments we also gathered data from human embryonal carcinoma cells (NCCIT and NTERA2).

ESCDb offers a summarized view of multiple pluripotency related datasets. Individual genes are described as a row in the output table. A colour-scheme helps to illustrate the potential regulatory relations between genes. In the gene-expression datasets often more than one probe-set represent a gene and we treat each individual probe-set individually. We kept the same order of probe-sets in the output table for easier comparisons between probe-sets in all available datasets. Further details are given in numerical form when a given cell of the table is pointed with a cursor. The database can be queried with any widely used gene or protein identifier or Gene Ontology terms.

The current version of the database comprises gene expression data from 18 mouse transcription factor-targeting experiments for 14 known factors [3], [41], [42] (Oct4, Sox2, Nanog, n-Myc, c-Myc, Stat3, Suz12, Klf4, Zfx, Tcfcp2l1, Smad1, Ctcf, E2f1, Esrrb), 6 human transcription factor binding experiments [3], [14], [43] for the 3 main pluripotency regulators (OCT4, SOX2, NANOG), 9 mouse ES cell knock-down experiments for Oct4, Sox2 and Nanog [15], [17], [18], [44], [45] and 9 perturbation experiments (including knockdowns of OCT4, SOX2 and NANOG in EC cells and overexpression of GADD45G in EC cells), BMP4 and ACTIVIN A stimulated hES cells and FGF2 withdrawal from hES cells during culture [6], [46].

## Discussion

ChIP-based studies on the transcription factor OCT4 have been carried out by others [3], [14], [15]. However, none of these studies compared the peak regions identified using different detection programs. As demonstrated in this work, using the online available programs MAC2 and TAMALPAIS and an in-house implementation of a ratio distribution dependent interval analysis developed algorithm for peak discovery, the overlap of target genes identified between the single programs is below 50%. This means that a substantial proportion of potential binding sites would be lost by depending on one algorithm in isolation. TAMALPAIS and MAC2 seemed the best algorithms for true positive prediction, although they would not achieve AUC (Area Under ROC Curve) values beyond 0.7, using ROC-like curves (receiver operating characteristic curves) for diluted spike ins [18]. ROC-like curves plot sensitivity vs. filtering fraction at every threshold. On ROC curves, the True Positive Rate is plotted against the False Positive Rate calculated at each cut-off [47]. To compare two different methods, usually the area under these curves is computed. A random method would have an area equal to 0.5 and a perfect method would have and area equal to 1. True positive peaks might be represented by different complex peak shapes, which one algorithm alone would not detect and thus the approach presented here, combining different programs in a rank based score, potentially leads to a more complete target list.

We previously demonstrated that NCCIT cells are a useful model system for investigating pathways involved in maintaining self-renewal [6]. Thus, we wondered in how far NCCIT-derived OCT4 downstream target genes could be compared to human ES cell-derived OCT4 target genes [3] and target genes derived from another EC cell line- NTERA-2 [14]. The overlap we report here is below 10%. This is based on the different platforms and the different peak finding programs used, thus confirming that different programs identify overlapping but also distinct sets of target genes. Finally, the comparisons are valid only for a selected promoter region for which there is evidence that most binding events occur [3]. However they reveal potential functional binding events, which are associated with non-proximal promoter specific regions. We could not detect cell-type specific pathways correlating with OCT4 binding within EC and ES cells. Nonetheless, among the targets identified in this study and confirmed by other studies are key stem cell markers like NANOG, OCT4, SOX2 and HESX1, other homeodomain-containing proteins like NKX2-2, SIX1, HOXB4 and LHX5, transcription factors like ZIC4 and SP8 and enzymes like DUSP6 and PPP2R3A, which are potential candidates for either sustaining self-renewal and pluripotency or inducing differentiation in ES cells.

The HMG factor SOX2, is known to form a heterodimer with OCT4 which results in a protein-protein-DNA complex required for transcriptional regulation of genes such as Utf1, Fbx15, Sox2 and Nanog [48], [49], [50], [51]. Based on the plurality of interactions between HMG and POU class proteins and the co-evolution of HMG/POU DNA target sequences, this interaction is thought to be a fundamental mechanism for the developmental control of gene expression [52]. Furthermore, as shown for the Fgf4 promoter, the distance between the binding recognition sites of SOX2 and OCT4 seem to be crucial for synergistic activation [52]. We also observed that OCT4 and SOX2 motifs tend to have a closer distance between each other, independent of strand orientation (data not shown). We next posed the question if the close proximity of the binding recognition sites of SOX2 and OCT4 is a pre-requisite for the proper assembly of functional activation complexes. Our results suggest that there is no such correlation. This is based on the unveiling of 6 distinct modules of OCT4-regulated gene regulatory networks with genes within or between each module having distinct distances between the SOX2 and OCT4 binding motifs or even not having a SOX2 motif adjacent to that of OCT4 (Fig. 6). Based on these results, it seems that the SOX2-OCT4 motif or the close proximity of both motifs is not required for the majority of OCT4 regulated target genes. For these genes, octamer motifs might be more displaced from our peak regions and hint at protein-chromatin interactions, bringing different chromatin regions into close proximity.

Boyer and colleagues [3] revealed that approximately half of the promoter regions discovered by ChIP-on-chip analysis, occupied by OCT4 were also bound by SOX2 in human ES cells. In our analysis with human EC cells, using the *in silico*-derived SOX2 motif for target identification instead of peak regions, unveiled 108 SOX2-motif related putative binding sites out of 497 total binding sites and 161 binding sites linked to an OCT4 motif. However, this is only a fraction of the putative SOX2 binding sites identified in hES cells, thus suggesting distance related effects and/or other SOX2 motifs not discovered with our analysis. Additionally, one has to bear in mind that all thresholds defined for the OCT4 and SOX2 PWMs are arbitrarily set and therefore can only provide a prediction for a bona fide functional binding event, hence further experimental validation will be needed.

To identify binding modules, where the octamer element is not present, we also screened the 497 target genes for the presence of PORE or MORE motifs as an addition to target genes defined by module 4. We identified 4 putative target genes harbouring a PORE motif (module 5) and 10 target genes, which contained a MORE motif (module 6). Using a previously identified MORE (CTGCATATGCAT) motif within the *Bmp4* promoter, Kang et al. [53] verified an interaction between Oct4 and this genomic region and showed using mouse ES cells subjected to ionizing radiation that Oct4 occupancy was induced by stress. Based on these observations, it is tempting to speculate that maybe a subset of OCT4 targets harbouring the MORE motif might be associated with the modulation of stress responses.

Taken together, we provide a testable concept of distinct direct and indirect OCT4 binding patterns, depending on associated OCT4 related transcription factor binding sites. We used a similar approach applied by Segal and colleagues, to identify regulatory modules and their condition-specific regulators in yeast [54]. However, there was a difference in that we started our screen with potential transcription factor occupancy in relation to the presence of their specific binding sites. Recently, evidence in support of ChIP-on-chip based detection of indirect binding activities of transcription factors has been provided in an independent study by Gordan et al. [55]. Their method revealed that only 48% of targets could be explained by direct binding of the profiled transcription factors, while 16% could be explained by indirect binding. In addition to the approach presented here, the authors used *in vivo* nucleosome positioning. However they reported only a slight improve in the detection of indirect transcription factors and nucleosome data are not yet available for human EC or human ES cells. In addition, they suggested the probability of indirect transcription factor-DNA interaction when the motif of the profiled transcription factor is not significantly enriched in ChIP-on-chip data. However, this was not the case for the motif we uncovered for OCT4, but still around 66% of the enriched sequences did not contain OCT4 motifs, and one of the hypothesis of this study is that these sequences might still be valid candidates for putative indirect targets of OCT4.

**Figure 12:**
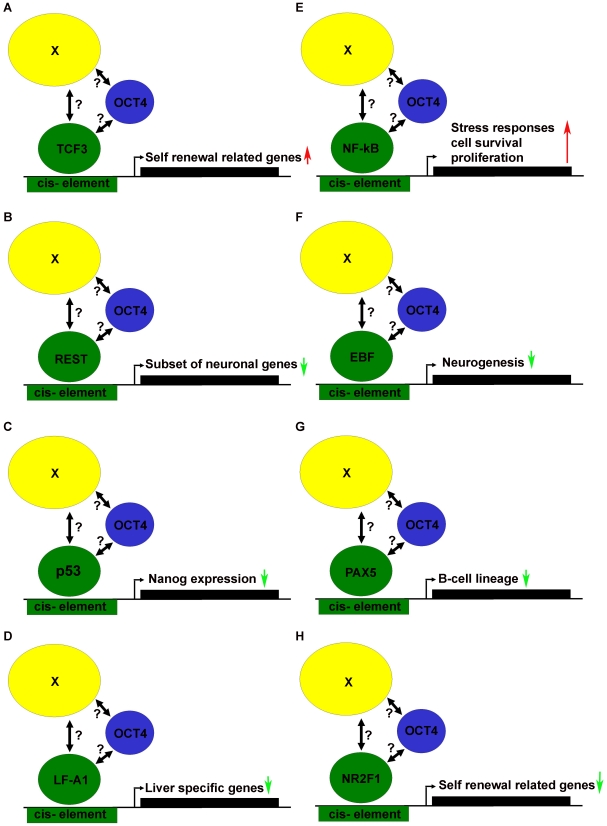
Hypothetical model based on module 4 of how OCT4 could be involved in regulating its target genes via non-direct DNA binding. OCT4 might be recruited by a mediator complex (X), which has additional affinity for the discussed transcription factors (A – H). Alternatively, there might be a direct interaction between OCT4 and the transcription factor(s) (indicated by ?), which might then potentially bind to the identified *in silico* cis elements. Arrows: Red- induction and green- repression of transcription of the respective target genes.

As a provocative thought, is there an OCT4 regulatory module specific for maintaining the self-renewal circuitry, or specific for suppression of the induction of differentiation to distinct cell lineages by the recruitment of co-activators or repressors to the OCT4 transcriptional complex. In response to these questions, we present hypothetical schemes (Fig. 12) which are based on the *de novo* motif discovery analysis performed on the OCT4 indirect target genes postulated to be regulated under module 4 (Fig. 6 and 11A).

As illustrated in Fig. 12 A and B, OCT4 might form a distinct or even the same complex with TCF3 and REST to maintain positive-gene regulatory networks supporting self-renewal. Interestingly both genes are highly expressed in undifferentiated ES and EC cells and their expression declines upon differentiation. Furthermore, TCF3 has been assigned as an integral component of an interconnected autoregulatory loop, where OCT4, SOX2, NANOG and TCF3 occupy each and their own promoters in maintaining the self-renewal circuitry in embryonic stem cells [56]. REST, a transcriptional co-repressor has been shown in mouse ES cells to selectively repress transcription of a subset of neuronal genes [57].

Another protein complex that might promote self-renewal is composed of OCT4 and NF-kB (Fig. 12 E) in positively regulating gene networks in response to stress signals to activate cell survival and proliferation pathways [58]. Furthermore, the regulatory schemes depicted in Fig. 12 C–D, F–H, represents scenarios where the OCT4-bound complex might sustain self-renewal by inhibiting the differentiation inducing activities of transcription factors such as p53 [59], LF-A1 [60], EBF [61], PAX5 [62] and NR2F1 [63]. Unfortunately, experiments to test and confirm these hypotheses are beyond the scope of this study.

As a precautionary note, we cannot exclude the possibility that the OCT4-regulatory modules described here are just the tip of the iceberg and that with the adoption of an unbiased screen of OCT4 targets using ChIP-seq will reveal the complex nature of the self-renewal-gene regulatory network under the control of OCT4. A precedent for this is the identification in mouse ES cells of an extended network for pluripotency [48] and also indications that Oct4 can also bind to chimeric combinations of Oct4 half sites [64].

With respect to characterized potential downstream targets of OCT4, we were intrigued by a possible direct regulation of USP44, an important regulator of the spindle checkpoint. We uncovered a highly conserved OCT4 binding site within its proximal promoter and a significant decrease of the transcript level in OCT4 knockdown experiments [6], [9]. Furthermore, screening the online hESC expression atlas Amazonia [65], we uncovered a significant decrease of this transcript upon embryoid body-based differerentiation, and the level remains low in various somatic tissues. Based on these findings we propose that USP44 is a positive regulator of self-renewal in EC as well as ES cells and that this regulation could be mediated by its prominent role in regulating the spindle checkpoint during the cell cycle [66].

Another major finding emerging from this study is the identification of GADD45G - a regulator of the cell cycle at the G2/M transition [33] and also recently identified as a putative OCT4/PORE target gene [34]. We verified the presence of the OCT4 binding motif within its promoter, additionally, our array data revealed up-regulated expression of this gene upon siRNA-induced ablation of OCT4 function in both human EC and ES cells [9]. Furthermore, transient over-expression of GADD45G in NCCIT cells induced up-regulated expression of GADD45A as well as expression of genes associated with the cell cycle and differentiation processes. Interestingly, the expression level of CR6-interacting factor 1, shown to interact with the GADD45 family and modulate the cell cycle [67] did not change upon the over-expression of GADD45G, thus non-supportive of a feedback loop between the GADD45 family members and CR6-interacting factor 1. This coupled to the fact that there are indications in mouse ES cells that the transcription level of *Gadd45g* increases significantly upon differentiation [17], makes it tempting to speculate that OCT4 negatively regulates the transcription of GADD45G in order to maintain self-renewal in EC and ES cells.

Finally, in this era of high-throughput functional genomics and systems biology-driven research, which necessitates large datasets, there is a dire need for data integration platforms. To facilitate this, we have integrated our datasets along with existing related datasets from both human and mouse ES and EC cells to generate an Embryonic Stem Cell Database (ESCDb) which allows rapid and convenient access and comparisons between published datasets related to embryonic stem cell biology. We anticipate that this study will aid in increasing our meager understanding of self-renewal in ES, EC, iPS and cancer cells.

## Materials and Methods

### Cell culture

NCCIT cells were grown in high-glucose DMEM supplemented with 10% FCS (Biochrom, Berlin/Germany), 2 mM glutamine, and penicillin/streptomycin on conventional tissue culture plastic surfaces.

### ChIP-on-Chip

Human NCCIT cells were grown to a final count of 5×10^7^–1×10^8^ cells for each Immunoprecipitation. Cells were chemically crosslinked by the addition of one-tenth volume of fresh 11% formaldehyde solution for 10 min at room temperature. Cells were rinsed twice with 1× PBS and harvested using a silicon scraper and flash frozen in liquid nitrogen and stored at −80°C prior to use. Cells were resuspended, subjected to lysis buffers, and sonicated to solubilize and shear crosslinked DNA. Sonication conditions vary depending on cells, culture conditions, crosslinking, and equipment. We used a BRANSON 250 and sonicated at power 3 for 11:00 min with 30% Duty Cycle at 4°C while samples were immersed in an ice bath. The resulting wholecell extract (WCE) was incubated overnight at 4°C with 100 µl of Dynal Protein G magnetic beads that had been preincubated with 10 µg of OCT4 antibody (insert). Beads were washed five times with RIPA buffer and once with TE containing 50 mM NaCl. Bound complexes were eluted from the beads by heating at 65°C with occasional vortexing, and crosslinking was reversed by overnight incubation at 65°C. Whole-cell extract DNA (reserved from the sonication step) was also treated for crosslink reversal. Immunoprecipitated DNA and whole-cell extract DNA were then purified by treatment with RNase A, proteinase K, multiple phenol:chloroform:isoamyl alcohol extractions and precipitation with ethanol. Purified DNA was amplified using a one-stage random PCR protocol. For ChIP-on-chip assay three biological replicate ChIP experiments were performed. Labelling and hybridisation of ChIP-DNA was done by the NimbleGen company. Using the NimbleGen human promoter tiling arrays (HG18) we screened 6517 putative promoter regions more, with a median probe spacing of 100 bp, compared to the OCT4 ChIP-on-Chip done by Boyer et al. Though the chip was covering only 4250 bp, these probes were within the most abundant TF binding sites, using TRANSFAC [68].

### Bandshift assays

For the Bandshift assays, nuclear extracts were prepared from NCCIT cells, using the method of Dignam et al. [69], with the modifications of Rodda et al. [70], using double stranded-DNA oligonucleotides (INVITEK) labelled with Cy5 at the 5′termini of both strands (Table S4). For DNA binding reactions 4 µl (40 µg) of nuclear extract was added to a 40 µl reaction (final) containing 50 nM Cy5-labelled oligonucleotide and 5 µg poly-dGdC (Amersham). The final binding buffer composition was 60% with 1 µg/µl BSA. Where specified, 1 µM unlabelled double stranded competitor was also included prior to the addition of nuclear extracts. Where specified, 2 µl anti-OCT4 (sc-9081x, Santa Cruz) antibody was added. Binding reactions were resolved on pre-run 6% native PAGE gels in 0.5X TBE for overnight at 50 V. Gels were imaged directly using a Fujifilm FLA-5100-R scanner.

### Biotinylated DNA Pull-down of OCT4 targets

50 µl streptavidin conjugated Dynabeads (Dynal) were washed with PBS-BSA (PBS, pH 7.4, 0.1% BSA) for each sample. Biotinylated USP44 promoter fragment DNA (100 pmol) was incubated with the streptavidin beads for 4 h at 4°C with rotation. Dynabead·DNA complexes were extensively washed with PBS-BSA to remove unbound DNA. Beads were added to 1000 µg Nuclear Extract of NCCIT cells (in Buffer D: 20 mM HEPES, pH 7.9, 20% glycerol, 100 mM KCl, 0.83 mM EDTA, 1.66 mM dithiothreitol, 1% protease inhibitor mixture, 50 µl polyGdC and 300X scrambled oligo). Samples were incubated for 8 h at 4°C with rotation. Dynabead-DNA-protein complexes were separated using the Dynabead magnetic station and then washed three times with ice cold Buffer D, adding 300X scrambled oligos each time. Samples were transferred to fresh microfuge tubes prior to final wash to avoid eluting plastic bound proteins. Dynabead-DNA-protein complexes were eluted in SDS-reducing sample buffer by heating at 95°C. Duplicate samples were pooled and equal volumes loaded onto 10% polyacrylamide gels for SDS-PAGE. Samples were transferred to nitrocellulose membranes and subjected to Western blot analysis. Western blotting was performed according to standard procedures and using chemiluminescence detection (ECL – Amersham). Antibodies used were OCT4 (sc-8629) and PARP1 (sc-7150) both from Santa Cruz.

### Real-time PCR

RNA was reversely transcribed using MMLV (USB) and oligo-dT priming. Real-time RT-PCR was carried out on Applied Biosystems 7900 instrumentation in 20 µl reactions containing 10 µl of SYBR Green PCR mix (ABI), 0.375 µM of each primer, and diluted cDNA. All primer pairs used were confirmed to approximately double the amount of product within one cycle and to yield a single product of the predicted size. Primer sequences are provided in Table S4. Relative mRNA levels were calculated using the comparative Ct method (ABI instructions manual) and presented as % of biological controls. ACTB and GAPDH transcript levels were confirmed to correlate well with total RNA amounts and therefore used for normalisation throughout.

### Microarray analysis

In all microarray experiments, biotin-labelled cRNA was generated employing a linear amplification kit (Ambion #IL1791) with 300 ng of genomic DNA-free, quality-checked total RNA as input. Chip hybridisations, washing, Cy3-streptavidin (Amersham Biosciences) staining, and scanning was performed on the Illumina BeadStation 500 platform employing reagents and following protocols supplied by the manufacturer. cRNA samples were hybridised as biological triplicates on Illumina human-8 BeadChips. Due to an at least 20-fold feature redundancy quantitative expression data can be obtained (http://www.illumina.com/pages.ilmn?ID=5). All basic expression data analyses were carried out using the manufacturer's software BeadStudio 1.0. Raw data were background-subtracted and normalised using the “rank invariant” algorithm, by which negative intensity values may arise. These and values below the detection limit were arbitrarily set to the level of threshold detection (S = 20) in order to avoid nonsense values for expression ratios. Differentially expressed genes were required to change by at least 50% at P<0.01 according to an Illumina custom model [71].

### ChIP Real-Time PCR Analysis

Duplicates of each sample were analyzed in a quantitative PCR reaction using the Applied Biosystems 7900 sequence detector and QPCR SYBR Green PCR mix (ABI). Data was analyzed with a threshold set in the linear range of amplification. The cycle number that any particular sample crossed that threshold (Ct) was then used to determine fold difference (enrichment). Fold difference was calculated as 2^(Ct(input)-Ct(ChIP))^. Melting curves of each amplified sample indicated formation of a single product in all cases. All samples were analysed as duplicates.

### NimbleGen ChIP-on-Chip data analysis, Quality control and normalization

The NimbleGen human promoter tiling array utilized in this study is a two-array set. Three replicates of the ChIP vs. Input experiment were performed resulting in a total set of six arrays. Each array was analysed separately. Because NimbleGen did not deliver array images, the array images were reconstructed based on the intensity values using Bioconductors image function [72]. Additionally, the density distribution of the two labels Cy3 and Cy5 were examined by applying limmas plot densities function [73] and the differing density distributions indicate the need for normalization. Normalization was performed array-wise using Bioconductors quantile function. Further quality controls were performed by creating scatter plots and MA-Plots and by calculating Pearson correlation coefficients for raw and for quantile normalized data. Five of the six arrays had raw correlation coefficients (Cy3 vs Cy5) in the range of 0.91–0.94, however, correlation coefficients are always slightly higher after quantile normalization. The quality control of the sixth array revealed a technical problem specific to this chip. The re-constructed array images revealed considerable uneven dye distributions and the scatter-plot as well as the correlation coefficient of 0.16 meant that this array had to be omitted from further analyses. Correlation coefficients for replicates ranged from 0.76 to 0.8 among the ChIP samples and from 0.78–0.9 among the Input samples, suggesting our ChIP-on-Chip experiments were reproducible. The complete results of the quality control including array images are presented in Table S1.

### Data integration in the form of a database

These 31 experiments (18 mouse transcription factor-targeting experiments for 14 known factors [3], [41], [42] (Oct4, Sox2, Nanog, n-Myc, c-Myc, Stat3, Suz12, Klf4, Zfx, Tcfcp2l1, Smad1, Ctcf, E2f1, Esrrb), 6 human transcription factor binding experiments [3], [14], [43] for the 3 main pluripotency regulators (OCT4, SOX2, NANOG), 9 mouse ES cell knock-down experiments for Oct4, Sox2 and Nanog [15], [17], [18], [44], [45] and 8 perturbation experiments (including knockdowns of OCT4, SOX2 and NANOG in EC cells and GADD45G overexpression in EC cells), BMP4 and ACTIVIN A stimulated hES cells and FGF2 withdrawal from hES cells during culture [46] have been carried out in mouse and human and using different experimental platforms. Therefore we used Ensembl identificators and gene names to perform mappings between original datasets. We used g:Orth from the g:Profiler toolset to perform ortholog conversions between mouse and human database identifiers and g:Convert from the same toolset to translate identifiers used in one experiment to our common Ensembl identifier [26].

### *De novo* motif discovery

The new OCT4 seqlogo was generated by mapping the motifs that had Levenshtein distance, which measures the changes that have to be made (insertions, deletions, substitutions) to make two sequences equal at most 2 to ATGCAAAT OCT4 consensus sequences. We mapped all motifs back to all the peak regions, took the longest matches allowing at most 1 bp gap between two motifs from the input set. We then aligned these motifs and produced a PWM and a sequence logo.

In order to screen for putative transcription factor binding sites other than OCT4 and SOX2, we performed a *de novo* motif discovery analysis based on specific promoter regions of OCT4 target genes derived from those genes which were at least 2 fold differentially regulated in NCCIT cells. By taking the genomic positions of the identified peaks as a reference (that is to a peak score of at least 0.5), we assembled the sub-sequences underlying the peaks. The selected sub-sequences were used as input for the TAMO package, a *de novo* motif discovery framework [29] that incorporates AlignACE [74], MDScan [75] and MEME [76]. The motif discovery was performed following the given sample code except the clustering module. All obtained motifs were compared to each other by applying the *minaligndiff* function of the TAMO distribution and when motifs occur with an alignment difference <0.2, only the motif with the highest bit score is further considered. Secondly, we computed entropy of the dimer distribution of the motif sequence as a measure for the motif complexity. Motifs with complexity score <1.0 were discarded.

### Database matching of discovered motifs

The discovered motifs were compared against two existing databases of known motifs using the STAMP tool [30]. Motifs were compared against the TRANSFAC (v11.3) [65] and JASPAR (v3) [28] databases using the recommended default parameter settings.

### Peak finding algorithms

Three programs were adopted in this study:

#### Interval Analysis (brute-force)

Based on the quantile normalized data, for each oligonucleotide a fold-enrichment was calculated by dividing the signal intensity from the immunoprecipitated sample by the signal intensity of the whole-genome sample. For each array, the total ChIP/IP ratio distribution was examined in order to obtain array specific threshold values for the upper 0.01 and for the upper 0.05 quantile. A potential binding event is defined with respect to the estimated average fragment size of the sonicated genomic DNA (550 bp) in relation to the distance of oligonucleotides relative to the promoter regions of the examined TSSs (distances between oligonucleotides is 100 bp). Therefore, a potential binding event is defined as at least three oligonucleotides that fulfil the following criteria: a centre oligonucleotide has a ChIP/IP ratio within the upper 0.01 quantile of the total ratio distribution and one upstream and one downstream neighbour each within a distance of max. 1000 bp have a ChIP/IP ratio within the upper 0.05 quantile. All identified peaks were linked to the closest transcription start site (TSS), if one exists within a distance of 8 kb. Genomic positions of transcription start sites are based on Ensembl [77] and were downloaded via biomart [78].

#### MA2C

We used MA2C with standard settings for first normalizing our PairData files for each of the five experiments and thereafter searched for peaks [20]. Promoter 1 (3 replicates) had 269, 504, 460 peaks. Promoter 2 (2 replicates) had 1366 and 915 peaks. When we challenged MA2C with all three replicates simultaneously and used replicate function the program identified 830 peaks for promoter 1 and 1208 for promoter 2. When all three programs identify a peak close to a gene then the peaks found by MA2C tend to have the strongest OCT4 motif attached to it.

#### TAMALPAIS

We used a web version of the TAMALPAIS program for analysing already normalized files provided by NimbleGen [21]. TAMALPAIS searches for peaks in each array separately and lists as an output all peaks and their occurrences in different replicates. We chose the lowest stringency set of L4 for further analysis. We had for promoter1 1036 peaks in total, 54 that were found in all three replicates (max gap allowed between peaks is 50 bp), 93 that were found in two and 889 identified in only one replicate. For promoter two, we had only two biological replicates and for these we found 505 peaks, 32 of which were found in both and 419 that were found only in one replicate.

## Supporting Information

Document S1 Quality control of the OCT4 ChIP-chip data.
(2.75 MB DOC)

Table S1 Genes correlated with an OCT4 bound region in NCCIT, NTERA2 and H9 cells.
(0.04 MB XLS)

Table S2 Genes identified in the 6 different OCT4 binding modules.
(0.19 MB XLS)

Table S3 Regulated genes in NCCIT cells upon OCT4 and SOX2 knockdowns in relation to the 6 different OCT4 binding modules.
(0.03 MB XLS)

Table S4 Oligonucleotides and primers used in this study.
(0.03 MB XLS)
