# Supplementary material for: A Data Integration Approach to Mapping OCT4 Gene Regulatory Networks Operative in Embryonic Stem Cells and Embryonal Carcinoma Cells
Source: PLoS One. 2010 May 21;5(5):e10709. doi: 10.1371/journal.pone.0010709 (PMC2873957; doi:10.1371/journal.pone.0010709)
Supplement: Document S1 — Quality control of the OCT4 ChIP-chip data. (2.75 MB DOC) [file pone.0010709.s001.doc]

**Data analysis for Nimblegen ChIP-on-Chip data (6 chips).** Berlin, 2006-10-31

**Group Adjaye, Transcription factor Oct4.**

>SampleKey information:

| ORD_ID | CHIP_ID | DYE | DESIGN_NAME | DESIGN_ID | SAMPLE_LABEL | SAMPLE_SPECIES | SAMPLE_DESCRIPTION | TISSUE_TREATMENT | PROMOT_SAMPLE_TYPE |
| --- | --- | --- | --- | --- | --- | --- | --- | --- | --- |
| 4505 | 87866 | Cy3 | 2006-07-18_HG18_promoter_1of2 | 4227 | SOM008KG | Human (homo sapiens) | TOTAL III | Label with Cy3 | TOTAL |
| 4505 | 87866 | Cy5 | 2006-07-18_HG18_promoter_1of2 | 4227 | SOM008KE | Human (homo sapiens) | experimental III | Label with Cy5 | EXPERIMENTAL |
| 4505 | 89715 | Cy3 | 2006-07-18_HG18_promoter_2of2 | 4228 | SOM008KH | Human (homo sapiens) | total II | Label with Cy3 | TOTAL |
| 4505 | 89715 | Cy5 | 2006-07-18_HG18_promoter_2of2 | 4228 | SOM008KC | Human (homo sapiens) | experimental II | Label with Cy5 | EXPERIMENTAL |
| 4505 | 95313 | Cy3 | 2006-07-18_HG18_promoter_1of2 | 4227 | SOM008KJ | Human (homo sapiens) | Total I | Label with Cy3 | TOTAL |
| 4505 | 95313 | Cy5 | 2006-07-18_HG18_promoter_1of2 | 4227 | SOM008KD | Human (homo sapiens) | experimental I | Label with Cy5 | EXPERIMENTAL |
| 4505 | 95758 | Cy3 | 2006-07-18_HG18_promoter_2of2 | 4228 | SOM008KJ | Human (homo sapiens) | Total I | Label with Cy3 | TOTAL |
| 4505 | 95758 | Cy5 | 2006-07-18_HG18_promoter_2of2 | 4228 | SOM008KD | Human (homo sapiens) | experimental I | Label with Cy5 | EXPERIMENTAL |
| 4505 | 95760 | Cy3 | 2006-07-18_HG18_promoter_1of2 | 4227 | SOM008KH | Human (homo sapiens) | total II | Label with Cy3 | TOTAL |
| 4505 | 95760 | Cy5 | 2006-07-18_HG18_promoter_1of2 | 4227 | SOM008KC | Human (homo sapiens) | experimental II | Label with Cy5 | EXPERIMENTAL |
| 4505 | 95935 | Cy3 | 2006-07-18_HG18_promoter_2of2 | 4228 | SOM008KG | Human (homo sapiens) | TOTAL III | Label with Cy3 | TOTAL |
| 4505 | 95935 | Cy5 | 2006-07-18_HG18_promoter_2of2 | 4228 | SOM008KE | Human (homo sapiens) | experimental III | Label with Cy5 | EXPERIMENTAL |

**>Quality control:**

**Chip-wise:**

See the corresponding folders named by the ChipIDs for

- Reconstructed quality images

- Scatterplot and Pearson correlation coefficient, RAW data

- MA Plots for RAW data and the following normalization methods: MEDIAN, QUANTILE, VSN and LOESS

- Denity distributions for both channels for RAW and LOESS, MEDIAN normalized data.

MA-Plots:

x-axis=A=$\log_{2}\sqrt{Cy5*Cy3}$

y-axis=M =log_2_(cy5/cy3)

**Biological replica:**

See the directory ‘Biological_Replica’ for all pair-wise comparison between the channels of the chips.

**>Peaks:**

For the peak-finding the Quantile normalized date was used.

A peak is defined as a triple of oligonucleotides, where the ratio of the center oligonucleotide must be in the upper 0.01 quantile of all ratios and the two flanking oligonucleotides must each have a ratio within the upper 0.05 quantile of all ratios.

For each array, the corresponding subdirectories contain the following files:

- Quality Control images as included within this document
- The normalised data as ratios (*_Ratio_Quantile.txt)
- All identified peaks (*_635vs532-Ratio_Quantile.PeaksCombined)
- All identified and annotated peaks (*_635vs532-Ratio_Quantile.PeaksCombined.Annotated; annotated peaks are peaks where a TSS was fond within the range of -8kb and +8kb around the chromosomal peak position; TSS were taken from biomart ([www.biomart.org](http://www.biomart.org/)), human NCBI build 36)
- Preliminary images (*.jpg) for all annotated peaks (see the PeakImages directory)

A summary of all results is given in the file “Oct4_hECS_Peaks-Summary_Chavez.xls”

>**Chip_ID 87866**

**1. Reconstruction of the array images**

**
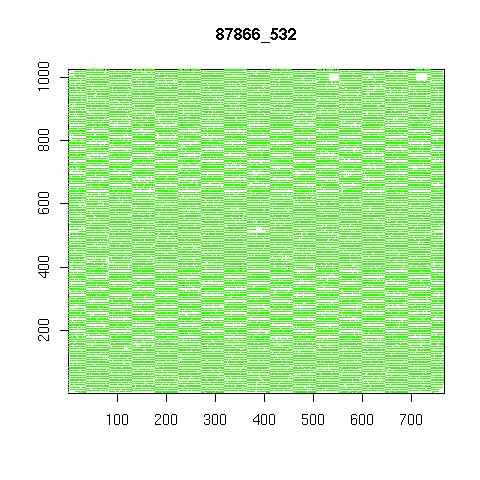

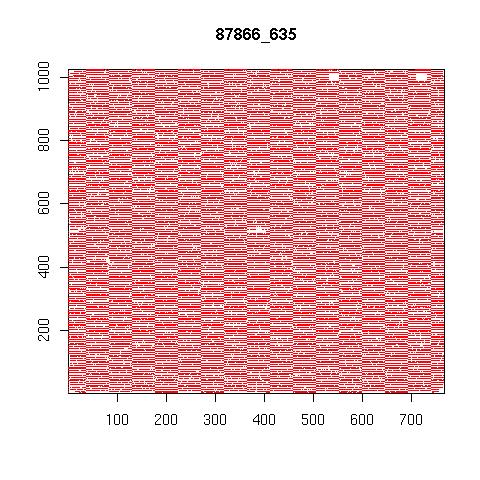
**

**2. Scatterplot RAW-Data (log) 3.1. MA-Plot RAW data**

**
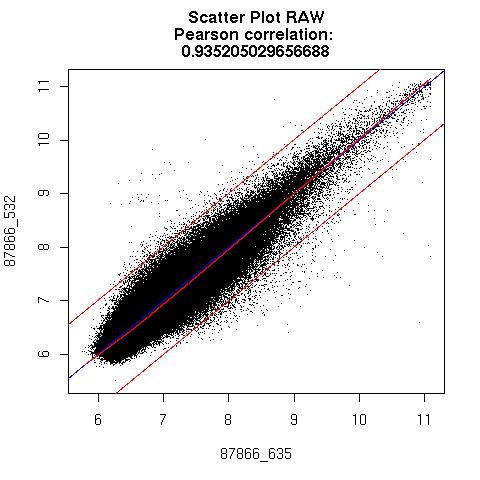

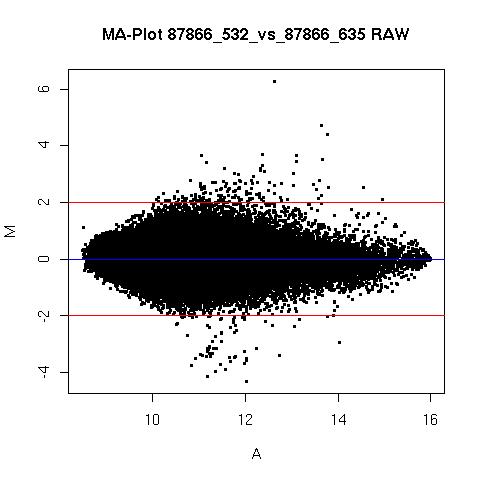
**

**3.2 MA-Plot MEDIAN normalized data 3.3 MA-Plot for VSN normalized data**

**
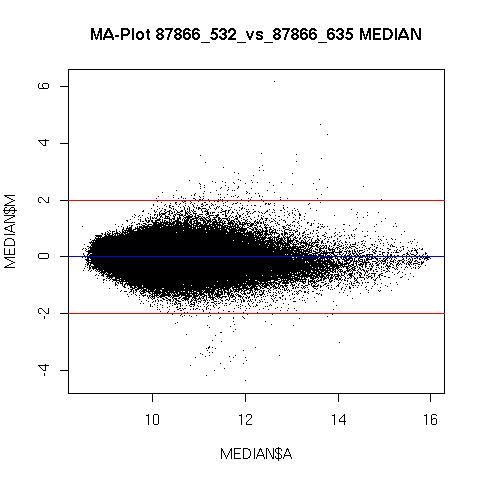

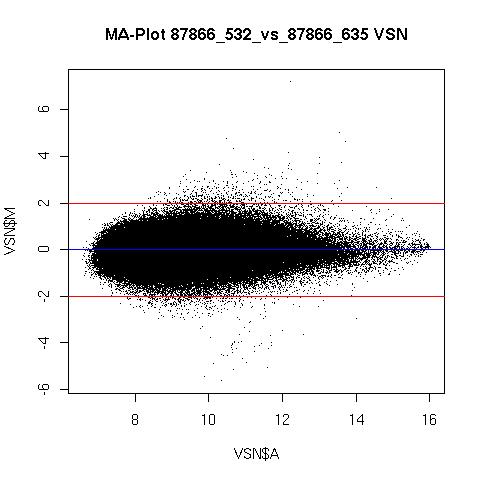
**

**3.4. MA-Plot LOWESS normalized data 3.5. MA-Plot QUANTILE normalized data**

**
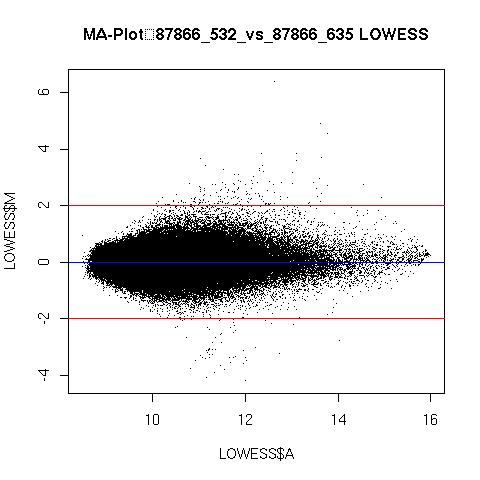

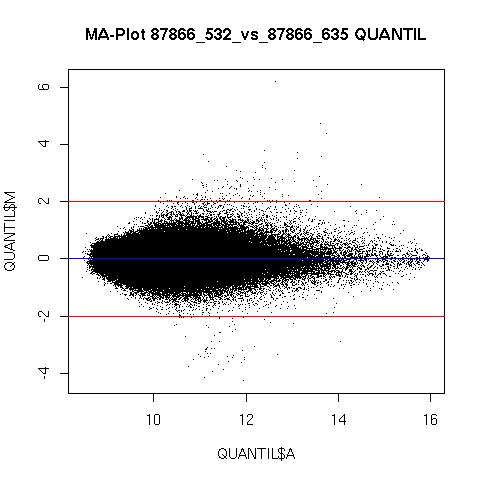
**

**4. Density distribution for the two channels**

**1. RAW**

**2. LOWESS**

**3. MEDIAN**

**
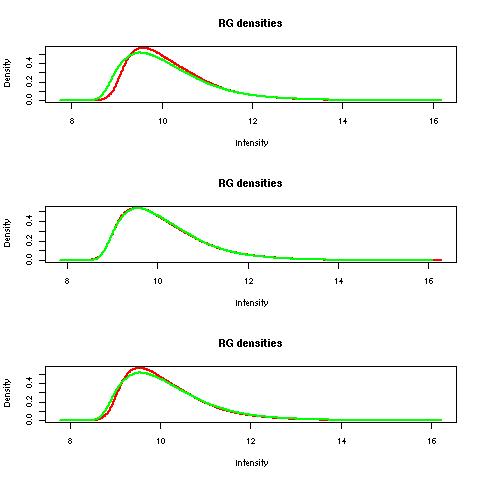
**

**>Chip_ID 89715**

**1. Reconstruction of the array images**

**
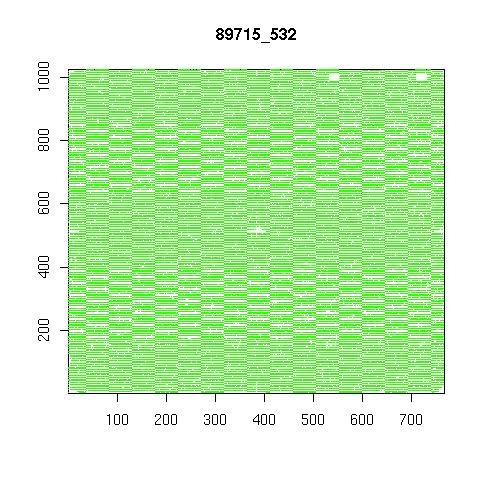

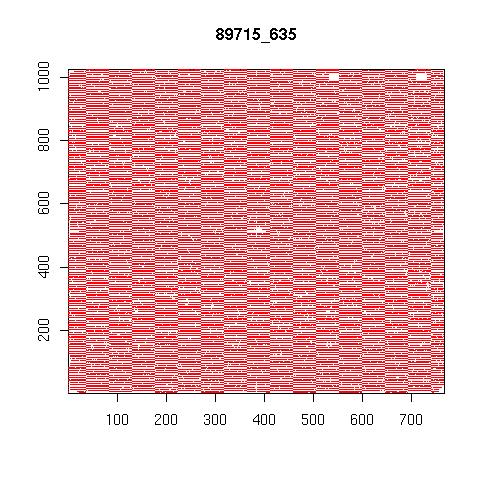
**

**2. Scatterplot RAW-Data (log) 3.1. MA-Plot RAW data**

**
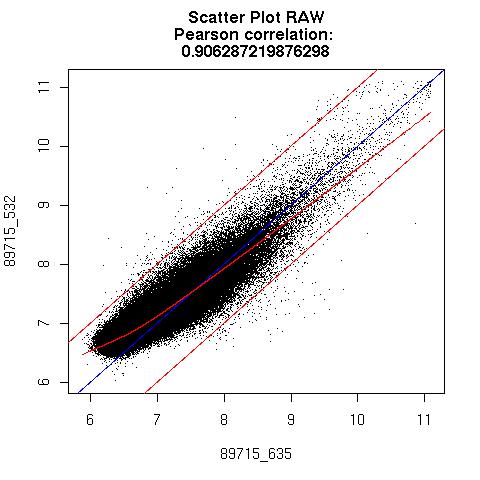

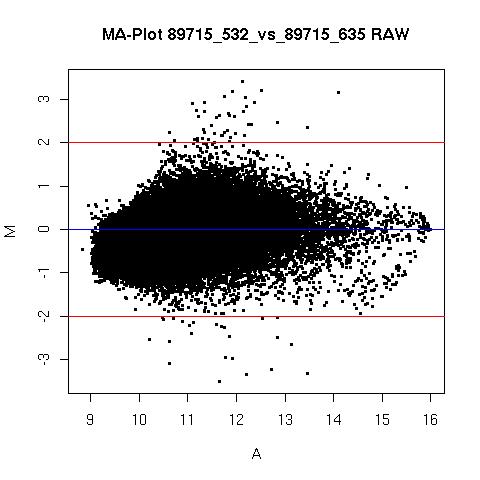
**

**3.2 MA-Plot MEDIAN normalized data 3.3 MA-Plot VSN normalized data**

**
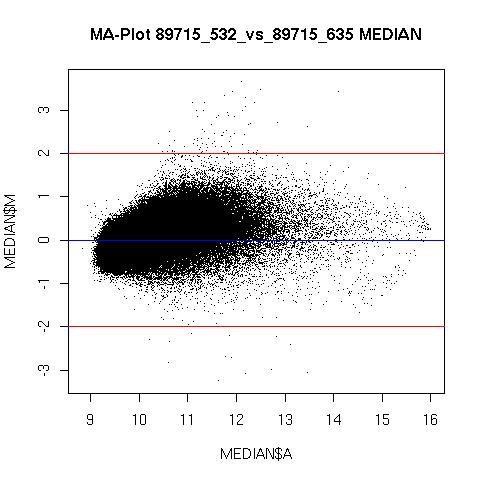

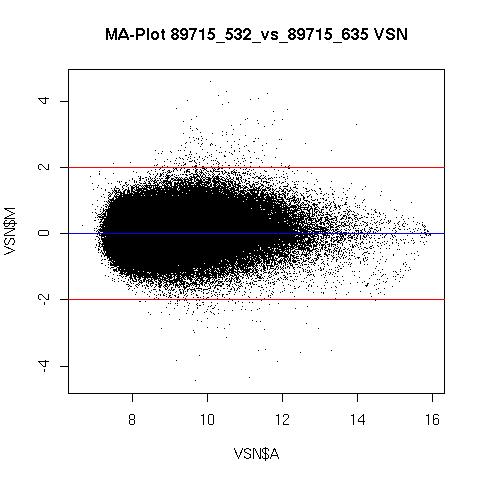
**

**3.4. MA-Plot LOWESS normalized data 3.5. MA-Plot QUANTILE normalized data**

**
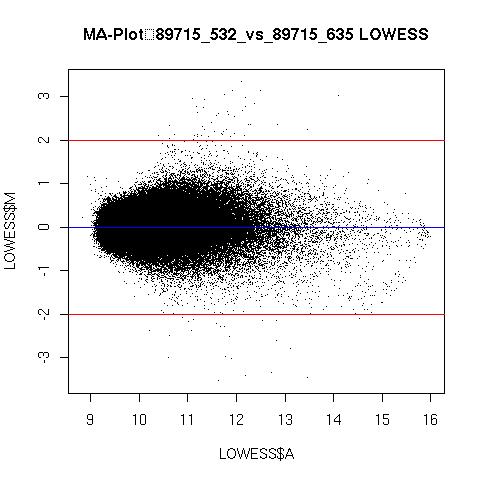

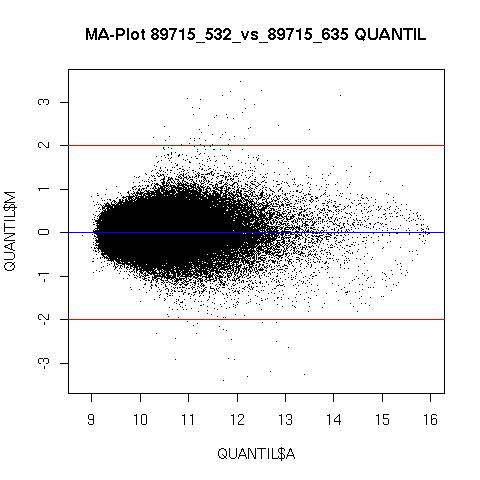
**

**4. Density distribution for the two channels**

**1. RAW**

**2. LOWESS**

**3. MEDIAN**

**
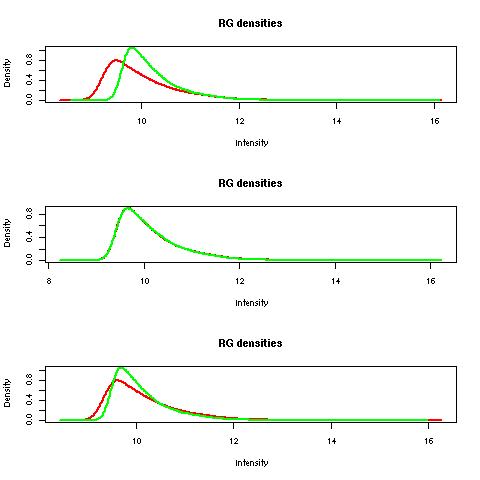
**

**>Chip_ID 95313**

**1. Reconstruction of the array images**

**
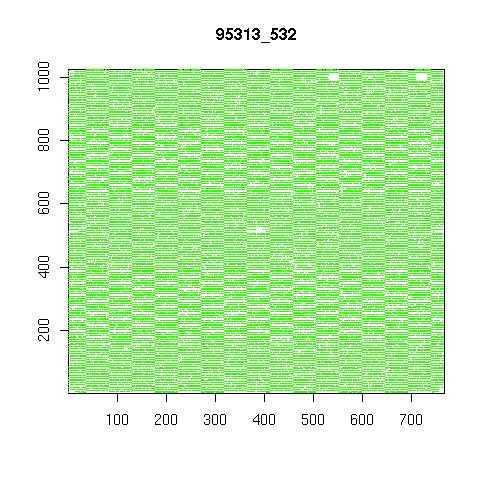

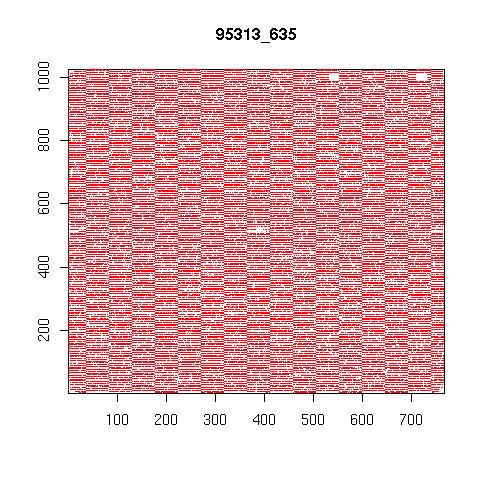
**

**2. Scatterplot RAW-Data (log) 3.1. MA-Plot RAW data**

**
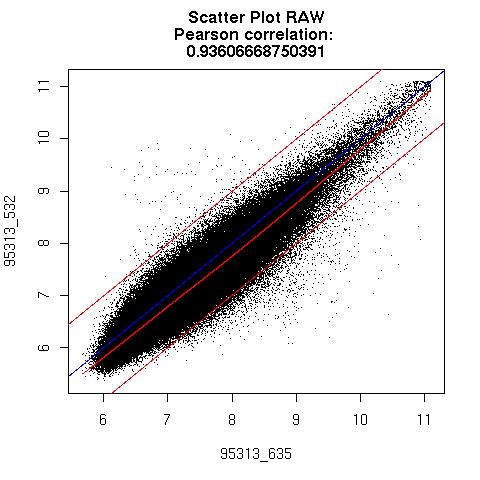

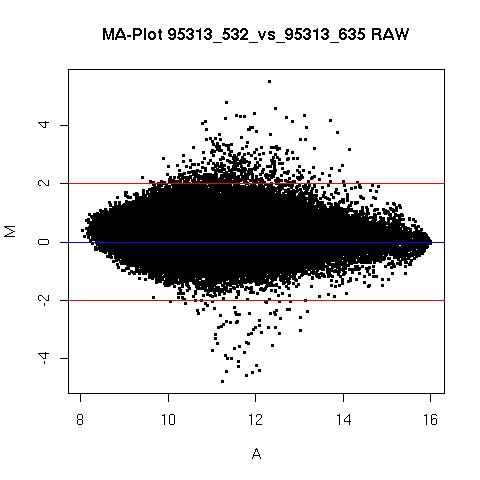
**

**3.2 MA-Plot MEDIAN normalized data 3.3 MA-Plot VSN normalized data**

**
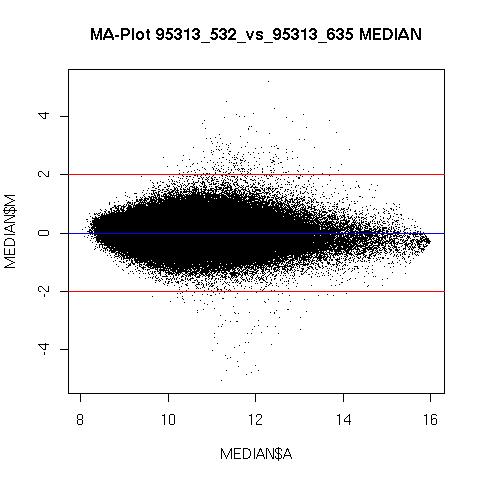

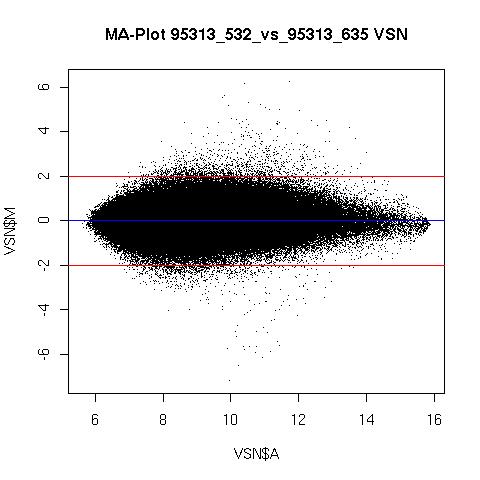
**

**3.4. MA-Plot LOWESS normalized data 3.5. MA-Plot QUANTILE normalized data**

**
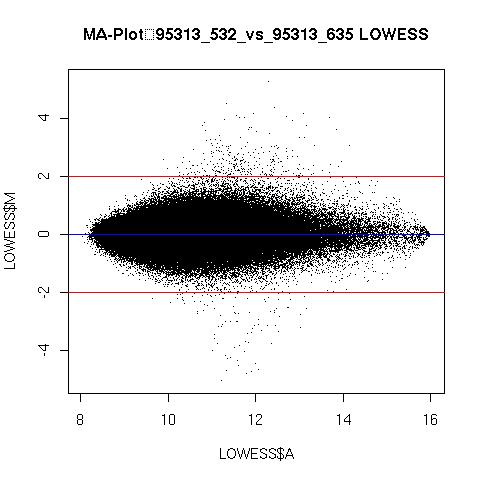

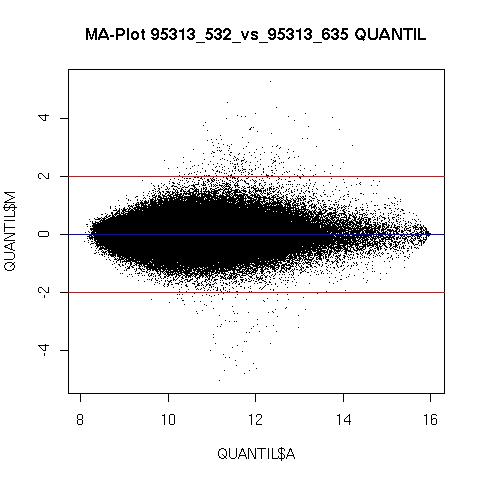
**

**4. Density distribution for the two channels**

**1. RAW**

**2. LOWESS**

**3. MEDIAN**

**
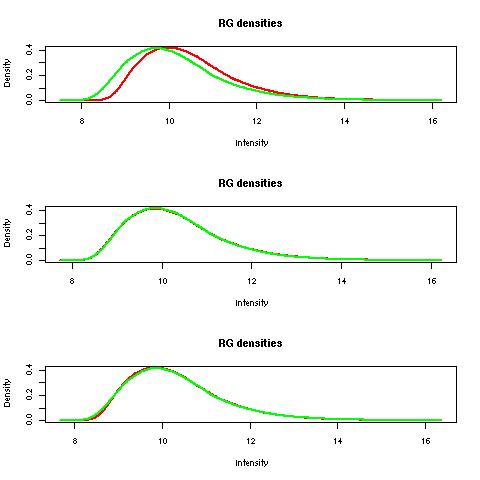
**

**>Chip_ID 95758**

**1. Reconstruction of the array images**

**
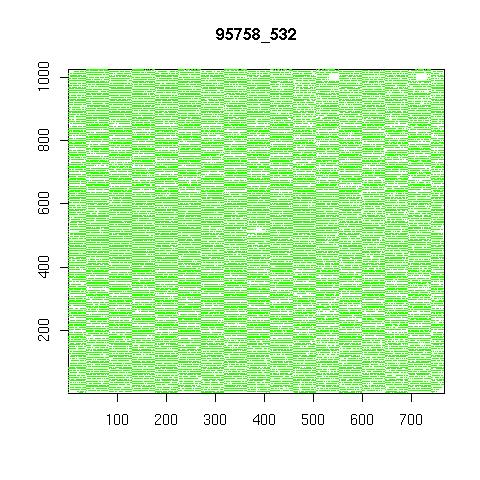

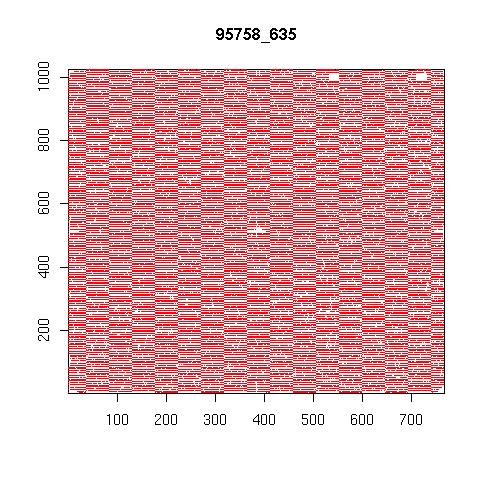
**

**2. Scatterplot RAW-Data (log) 3.1. MA-Plot RAW data**

**
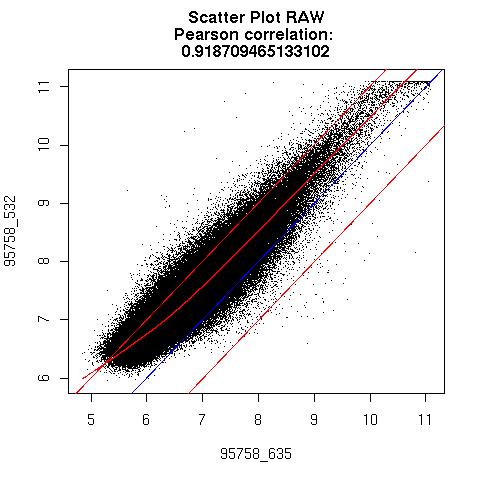

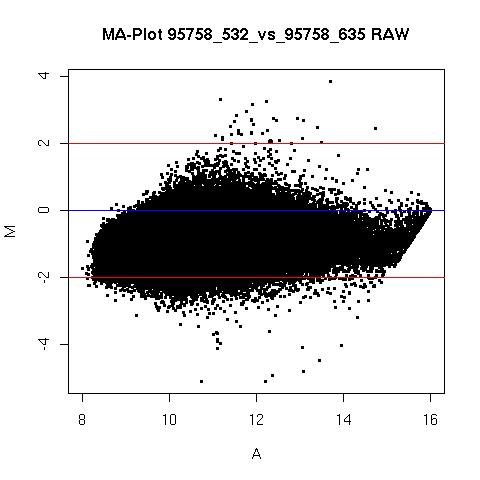
**

**3.2 MA-Plot MEDIAN normalized data 3.3 MA-Plot for VSN normalized data**

**
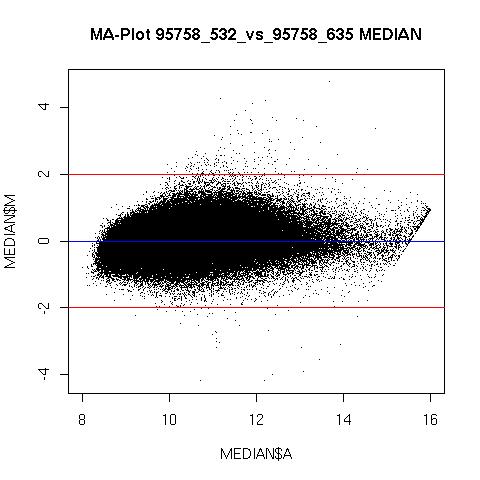

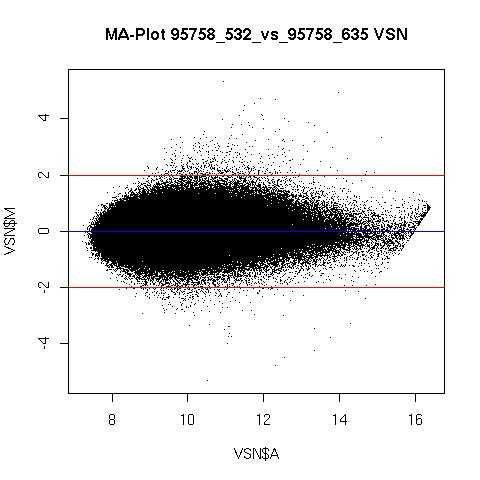
**

**3.4. MA-Plot LOWESS normalized data 3.5. MA-Plot QUANTILE normalized data**

**
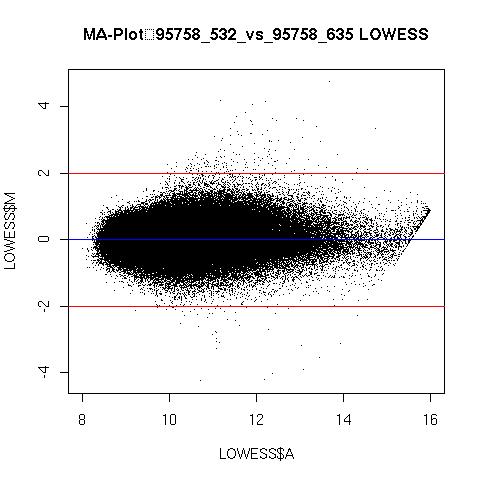

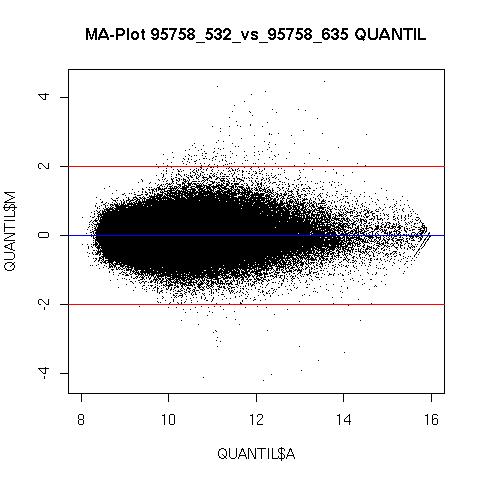
**

**4. Density distribution for the two channels**

**1. RAW**

**2. LOWESS**

**3. MEDIAN**

**
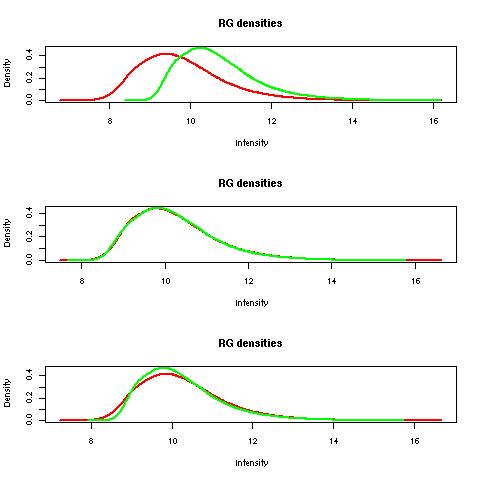
**

**Chip_ID 95760**

**1. Reconstruction of the array images**

**
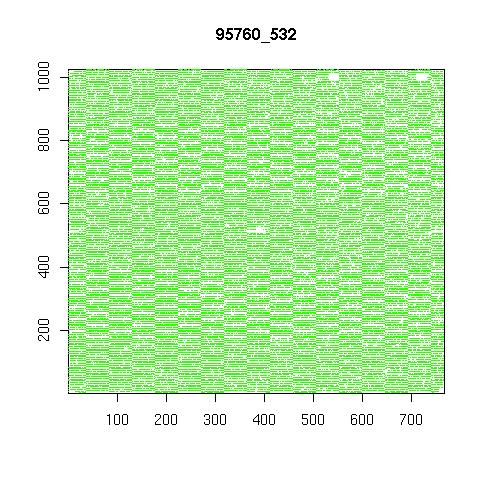

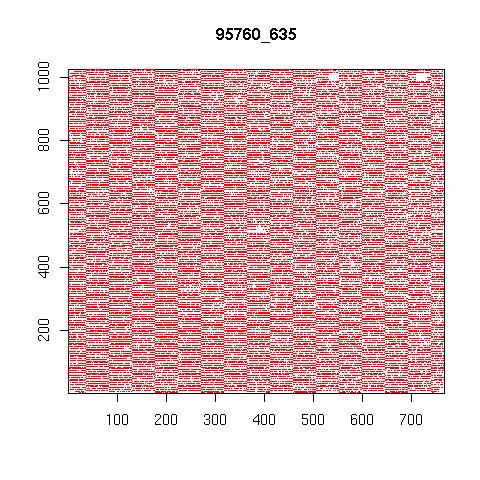
**

**2. Scatterplot RAW-Data (log) 3.1. MA-Plot RAW data**

**
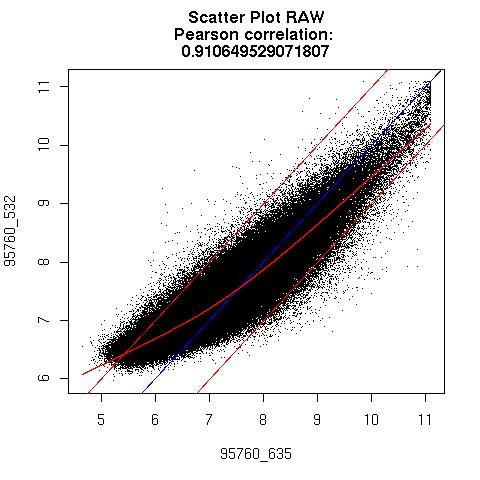

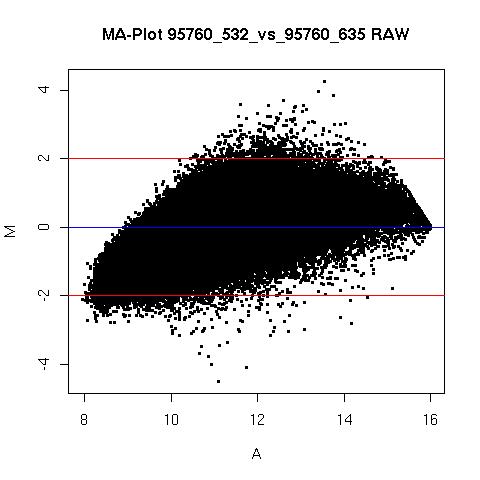
**

**3.2 MA-Plot MEDIAN normalized data 4.3 MA-Plot VSN normalized data**

**
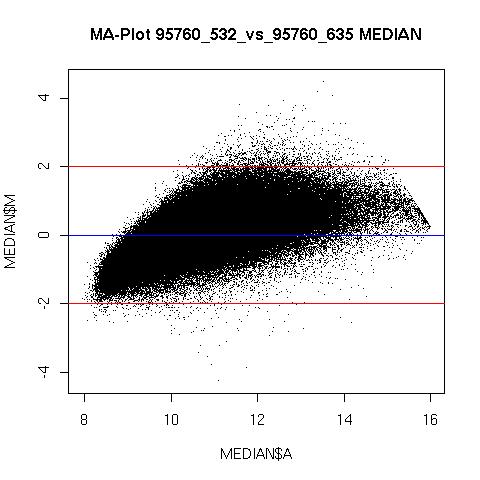

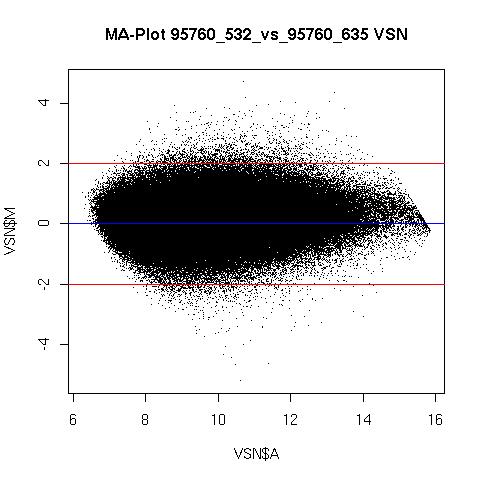
**

**3.4. MA-Plot LOWESS normalized data 3.5. MA-Plot QUANTILE normalized data**

**
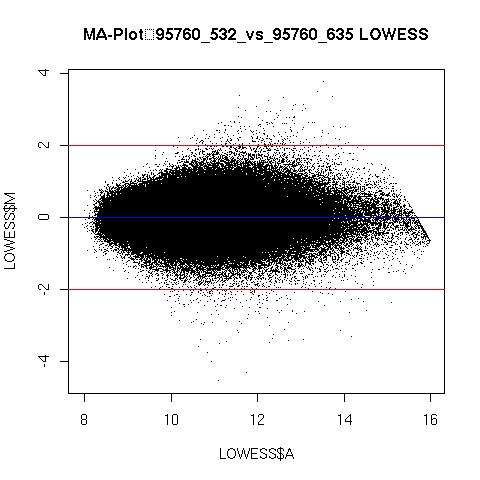

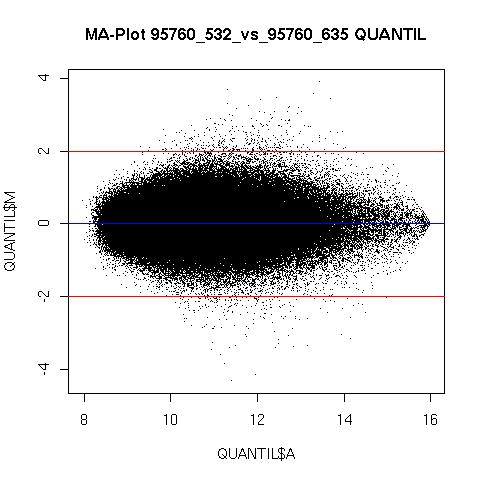
**

**4. Density distribution for the two channels**

**1. RAW**

**2. LOWESS**

**3. MEDIAN**

**
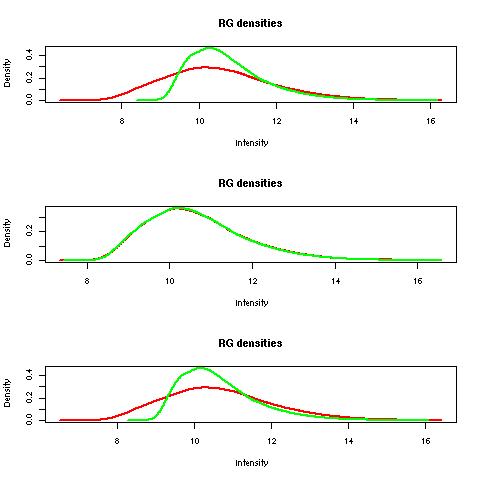
**

**>Chip_ID 95935**

**1. Reconstruction of the array images**

**
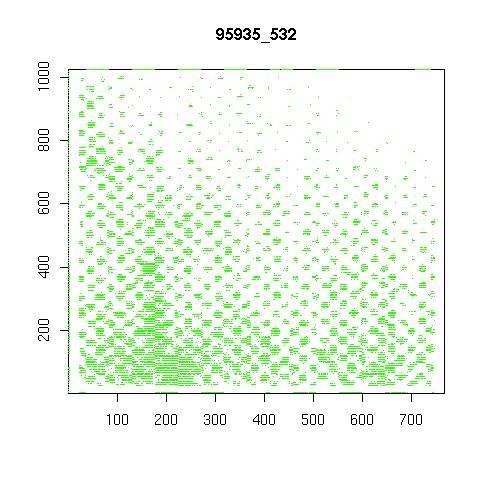

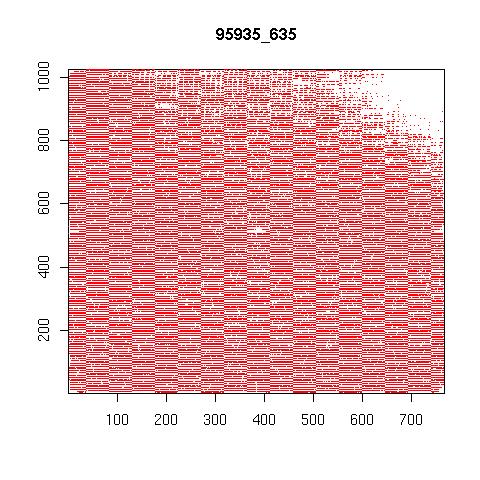
**

**2. Scatterplot RAW-Data (log) 3.1. MA-Plot RAW data**

**
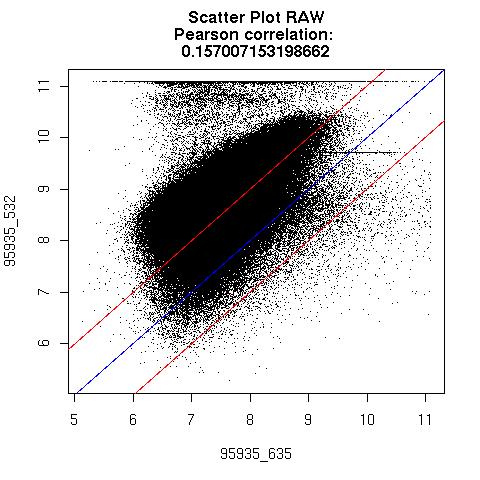
 NOT available.**

**3.2 MA-Plot MEDIAN normalized data 3.3 MA-Plot VSN normalized data**

**NOT available. NOT available.**

**3.4. MA-Plot LOWESS normalized data 3.5. MA-Plot QUANTILE normalized data**

**NOT available. NOT available.**

**5. Density distribution for the two channels**

**1. RAW**

**2. LOWESS**

**3. MEDIAN**

**NOT available.**

**Biological Replica:**

**1.2006-07-18_HG18_promotor_1of2. Cy3 (channel 532)**

**87866_532 vs. 95313_532 87866_532 vs. 95760_532**

**
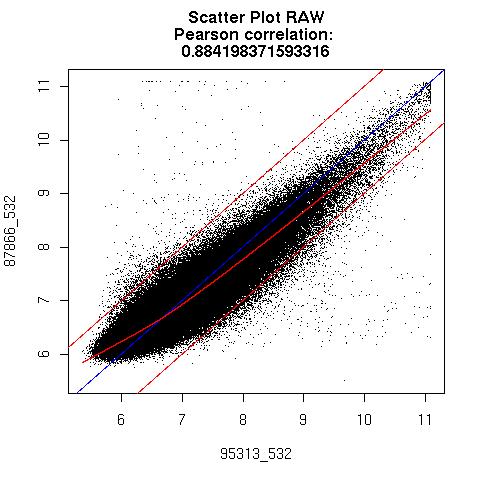

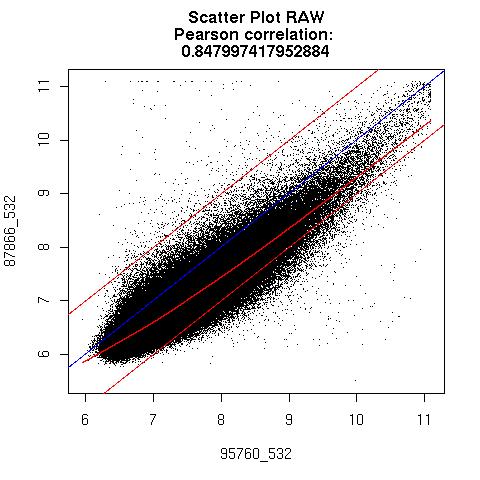
**

**95313_532 vs. 95760_532**

**
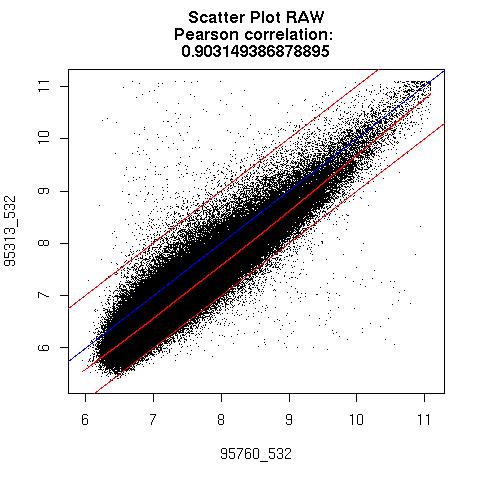
**

**2.2006-07-18_HG18_promotor_2of2. Cy3 (channel 532)**

**89715_532 vs. 95758_532 89715_532 vs. 95935_532**

**
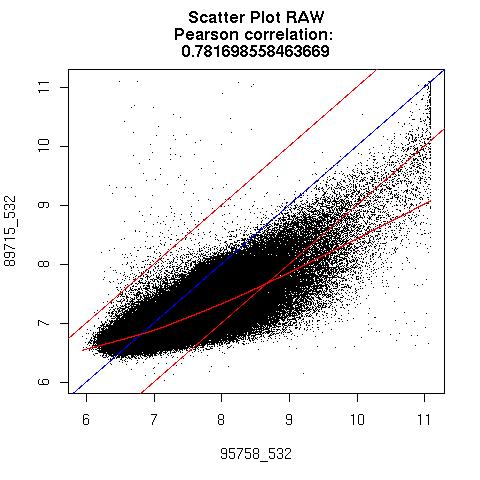

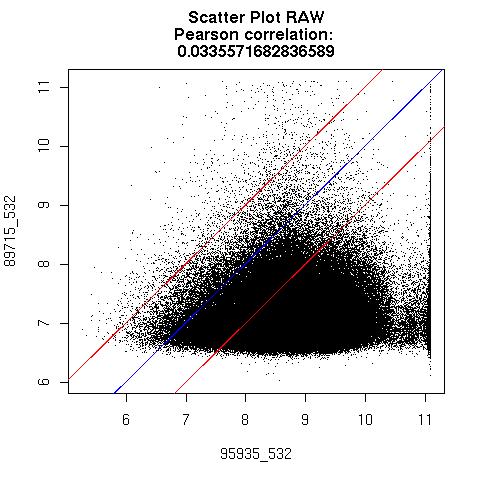
**

**95758_532 vs. 95935_532**

**
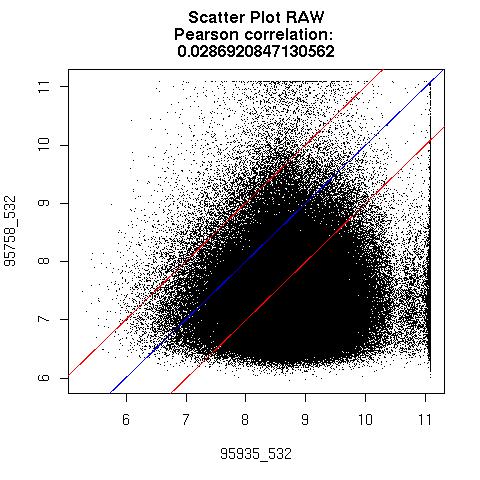
**

**1.2006-07-18_HG18_promotor_1of2. Cy5 (channel 635)**

**87866_635 vs. 95313_635 87866_635 vs. 95760_635**

**
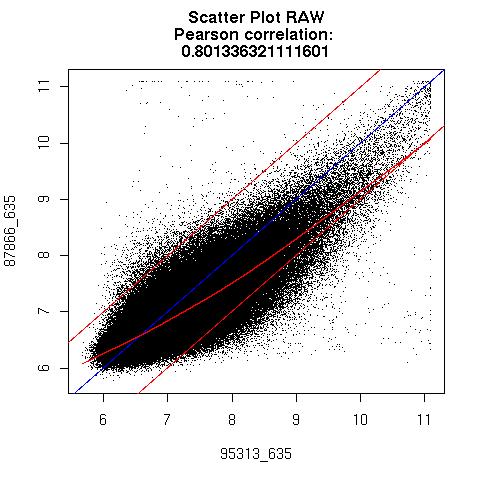

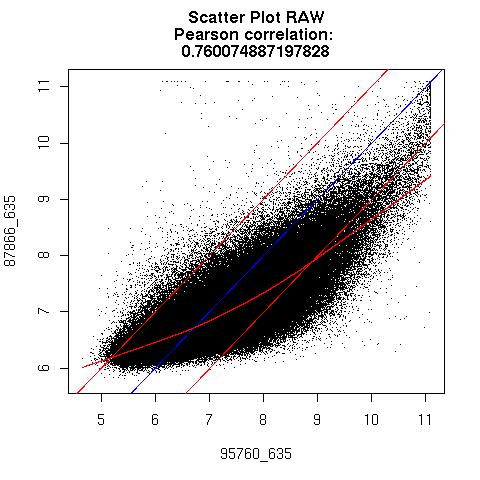
**

**95313_635 vs. 95760_635**

**
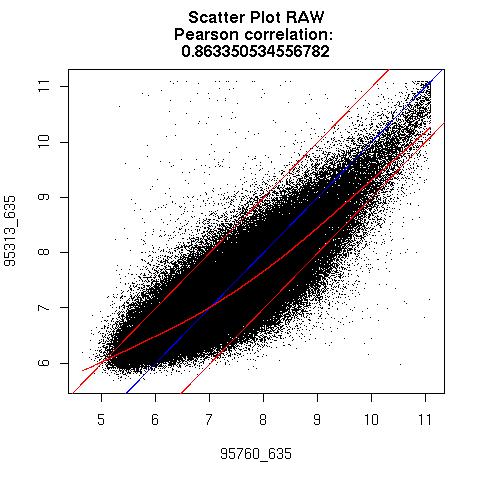
**

**2.2006-07-18_HG18_promotor_2of2. Cy5 (channel 635)**

**89715_635 vs. 95758_635 89715_635 vs. 95935_635**

**
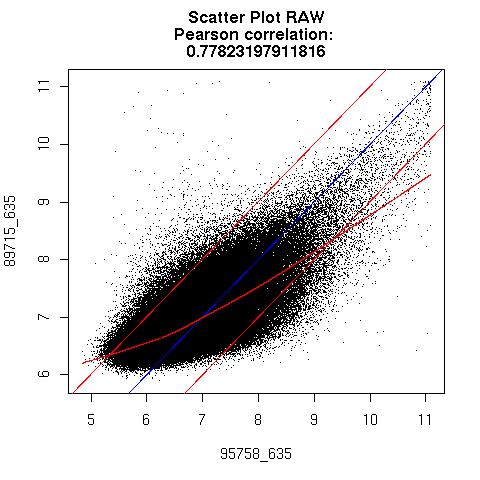

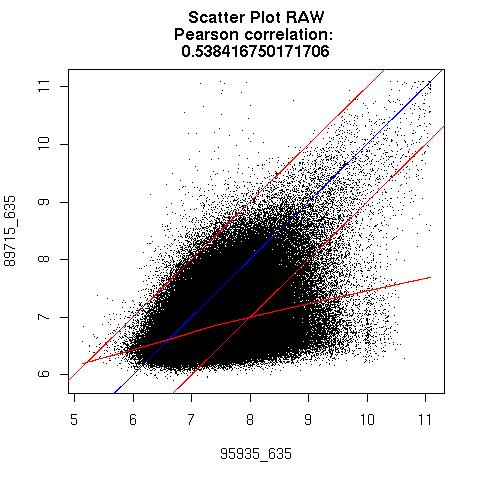
**

**95758_635 vs. 95935_635**

**
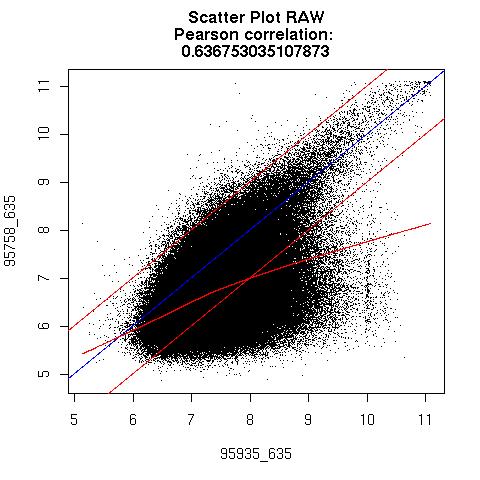
**
